# StemBond hydrogels control the mechanical microenvironment for pluripotent stem cells

**DOI:** 10.1038/s41467-021-26236-5

**Published:** 2021-10-21

**Authors:** Céline Labouesse, Bao Xiu Tan, Chibeza C. Agley, Moritz Hofer, Alexander K. Winkel, Giuliano G. Stirparo, Hannah T. Stuart, Christophe M. Verstreken, Carla Mulas, William Mansfield, Paul Bertone, Kristian Franze, José C. R. Silva, Kevin J. Chalut

**Affiliations:** 1 Wellcome-MRC Cambridge Stem Cell Institute, University of Cambridge, Puddicombe Way, Cambridge, CB2 0AW UK; 2 Cavendish Laboratory, Department of Physics, University of Cambridge, Cambridge, CB3 0HE UK; 3 Department of Physiology, Development and Neuroscience, University of Cambridge, Downing Street, Cambridge, CB2 3DY UK; 4 Institute of Medical Physics, Friedrich-Alexander University Erlangen-Nuremberg, 91052 Erlangen, Germany; 5 Max-Planck-Zentrum für Physik und Medizin, 91054 Erlangen, Germany; 6 Center for Cell Lineage and Atlas, Guangzhou Laboratory, Guangzhou International Bio Island, 510005 Guangzhou, Guangdong Province China; 7 Present Address: Department of Medicine, Alpert Medical School, Brown University, Providence, IR USA

**Keywords:** Biomaterials - cells, Stem-cell biotechnology, Embryonic stem cells, Reprogramming

## Abstract

Studies of mechanical signalling are typically performed by comparing cells cultured on soft and stiff hydrogel-based substrates. However, it is challenging to independently and robustly control both substrate stiffness and extracellular matrix tethering to substrates, making matrix tethering a potentially confounding variable in mechanical signalling investigations. Moreover, unstable matrix tethering can lead to poor cell attachment and weak engagement of cell adhesions. To address this, we developed StemBond hydrogels, a hydrogel in which matrix tethering is robust and can be varied independently of stiffness. We validate StemBond hydrogels by showing that they provide an optimal system for culturing mouse and human pluripotent stem cells. We further show how soft StemBond hydrogels modulate stem cell function, partly through stiffness-sensitive ERK signalling. Our findings underline how substrate mechanics impact mechanosensitive signalling pathways regulating self-renewal and differentiation, indicating that optimising the complete mechanical microenvironment will offer greater control over stem cell fate specification.

## Abstract (web summary)

The independent control of substrate stiffness and tethering of extracellular matrix to substrates for mechanical signalling investigations remains challenging. Here the authors present StemBond hydrogels, with stable ECM tethering that can be varied independently of stiffness, and use these to modulate the function of mouse and human pluripotent stem cells.

## Introduction

The physical properties of the cellular microenvironment, such as substrate stiffness and adhesiveness, have a strong influence on stem cell function, including maintenance and differentiation^1–3^. To control substrate stiffness in vitro, polyacrylamide (PAAm) hydrogels have been used extensively because their stiffness can be varied over several orders of magnitude within the physiological range, and their surface can be functionalised by the tethering of extra-cellular matrix (ECM) proteins. In one of the most widely used approaches in the field, polymerised PAAm hydrogels are treated with a hetero-bifunctional crosslinker, the most commonly employed one being sulfo-SANPAH, though alternative methods also exist^4–6^. UV-activated sulfo-SANPAH enables binding of protein to the surface of the hydrogels. Sulfo-SANPAH-treated PAAm hydrogels have been successfully used, for example, to study the impact of substrate stiffness on regulating fate choices of mesenchymal stem cells^7,8^. There have also been a handful of studies using these substrates to culture cells from soft tissue^9,10^. However, as we show here, cells—such as mouse embryonic stem cells (mESC)—that do not develop strong focal adhesions^11^ tend to detach from the substrates after a short time in culture. This limitation of commonly used PAAm substrates constitutes a significant bottleneck for studies in stem cell mechanobiology.

The detachment of cells from PAAm substrates could be due to a loss of ECM–substrate coupling, which is mediated by the reaction between the PAAm and the chosen crosslinker. For example, in the case of sulfo-SANPAH, a nitrophenyl azide group couples to acrylamide chains upon photoactivation. The non-specific nature of this reaction and the highly reactive nature of the intermediate photoactivated species render the binding to the substrate highly variable and difficult to control. Furthermore, it has been suggested that the effective crosslinker density depends not only on sulfo-SANPAH concentration, but also on hydrogel pore size^12^. The importance of pore size has been debated given that adult stem cells grown on substrates above 4 kPa are functionally insensitive to changes in ECM tethering density^13^. It is unclear whether that also holds true for stem cells cultured on much softer substrates below 1 kPa, as is typically done with cells from soft tissue such as embryonic or neural tissue^14,15^.

Embryonic stem cells are characterised by their ability to indefinitely self-renew in a pluripotent, undifferentiated state when in the presence of the appropriate soluble signals regulating key pluripotency pathways. mESCs can be maintained as a homogeneous population in a naïve, i.e. fully uncommitted, state by the dual inhibition (“2i”) of the GSK3β and the MEK/ERK pathways^16,17^. Alternatively, they can be maintained as a more heterogeneous population with a mix of naïve and more differentiated cells in serum-containing medium supplemented with the cytokine leukaemia inhibitory factor (LIF), an activator of JAK/STAT pathway (serum + LIF medium)^18–20^. Interestingly, all of the three aforementioned pathways—GSK3β, MEK/ERK and JAK/STAT—have been shown to be mechanosensitive in other cell types^12,21–24^. Moreover, a few studies have suggested that mESCs could sense and respond to substrate adhesiveness^25^ and stiffness^9,26,27^. However, these studies were performed either on plastic substrates or on sulfo-SANPAH functionalised soft substrates, neither of which offered good simultaneous control of stiffness and ECM tethering. As a result, exactly how the mechanical microenvironment impacts the pathways regulating pluripotent stem cell self-renewal or loss of pluripotency still remains to be elucidated.

To facilitate stem cell culture on soft substrates below 1 kPa, we sought an alternative PAAm functionalisation approach that would allow us to better control ECM tethering in a physiological range of hydrogel stiffness. We opted to incorporate a co-factor in the PAAm precursor solution that allows subsequent covalent binding of ECM proteins, similar to previous  demonstrations^28,29^. This approach allows controlling the density of ECM tethering points with the concentration of co-factor, while the stability of the functionalisation is ensured by the high specificity of the coupling reaction, irrespective of substrate stiffness.

In this work, we leverage our functionalisation method to obtain a range of substrates, which we call ‘StemBond’ hydrogels, for stem cell culture and investigations of mechanical signalling. To demonstrate how StemBond hydrogels can be applied for well-controlled investigations of mechanical signalling in stem cell self-renewal, we use mESCs as a model of weakly adherent cells. We show that controlling the strength of ECM tethering promotes the stability of the ECM layer and support the stable maintenance of both mESCs and human pluripotent stem cells (hPSCs), offering a tool to study the role of substrate, ECM and mechanics in stem cell self-renewal. We study the impact of substrate stiffness in mESCs and found that soft StemBond hydrogels allow self-renewal even in minimal medium conditions (i.e. a single chemical inhibitor) in which mESCs would normally differentiate. We additionally found that the efficiency of mouse epiblast-like stem cell reprogramming into naïve pluripotency was improved on soft substrates. We finally examine potential mechanosensitive signalling pathways regulating self-renewal in mESCs.

## Results

### StemBond hydrogels have controlled stiffness and ECM tethering strength

**Fig. 1:**
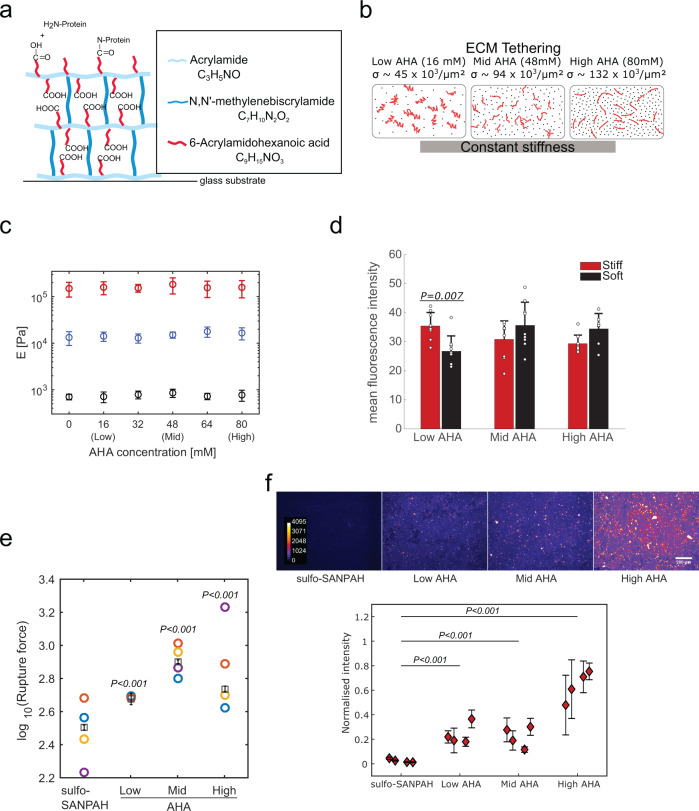
StemBond hydrogels provide control over ECM density and stability independent of stiffness. **a** Sketch of StemBond hydrogels. PAAm are synthesised with co-factor 6-Acrylamidohexanoic acid (AHA). The AHA chains terminate with a carboxyl group which then binds primary amines of ECM proteins. **b** Schematic of variations in ECM tethering strength with AHA concentrations. The estimated values of the surface density *σ* of carboxyl groups (black dots) is given for three different concentrations (see the “Methods” section). Each ECM protein fibre (red lines) can create many covalent bonds so different AHA concentrations will lead to different tethering strengths while not affecting the stiffness. **c** Young’s modulus of StemBond hydrogels with different proportions of acrylamide, bis-acrylamide and AHA co-factor was measured using AFM. The modulus in Pa is reported (mean ± standard deviation) (30 indentations/gel over 5 independent samples) for soft (black), intermediate (blue) and stiff (red) hydrogels. **d** Quantification of protein coverage on StemBond hydrogels, estimated by coating gels with FITC-labelled BSA and measuring the mean fluorescence intensity at the gel surface (*n* = 2–3 fields of view for three independent samples). Bars show mean ± standard deviation. *P*-values computed using a one-way ANOVA with Tukey–Kramer’s multiple comparison test. **e** Rupture force (log_10_) from significant binding events between fibronectin bound onto hydrogels and an AFM probe coated with anti-Fibronectin antibody. ‘○’: average of *n* = 3 or 4 independent experiments; ‘□’: overall mean; bars: standard error. *P*-values correspond to pairwise comparison to sulfo-SANPAH substrates from two-way ANOVA linear model. **f** Rhodamine-Fibronectin layer on different stiff substrates after 3 days of MEFs culture. (Top) Holes in the ECM layer are sites of ECM remodelling by MEFs. (Bottom) Mean ± standard deviation of fluorescence intensity for four replicates (*n* = 12–17 frames/data point taken over two independent experiments). Mean intensities were normalised for each batch and background was subtracted (measured on uncoated substrates) before averaging. *P*-values computed using a one-way ANOVA with Tukey–Kramer’s multiple comparison test. In panels **e** and **f**, all hydrogels were coated with 200 µg ml^−1^ fibronectin. Source data are provided as a Source Data file.

In order to establish an optimal mechanical microenvironment for pluripotent stem cells, and to enable their stable substrate attachment, we adapted a specific PAAm hydrogel protocol similar to what was suggested in refs. ^28,29^. We started from a precursor solution for a standard PAAm hydrogel below 1 kPa, approaching the low stiffness of the pre-implantation embryo from which mESCs are sourced (varying between 200 and 600 Pa^30^). We added to the precursor solution the co-factor 6-Acrylamidohexanoic acid (AHA), which can bind to acrylamide chains without crosslinking two acrylamide chains together. The terminal carboxyl groups of the AHA serve as anchorage points for covalent ECM protein binding by first forming an amine-reactive ester through a carbodiimide reaction. As with the widely used sulfo-SANPAH, this ester can react with any primary amine of, for example, lysine chains, to tether ECM proteins to the surface (Fig. 1a). However, this method allows comparatively better control over the binding and surface density of the carboxyl groups because the AHA chains polymerise with acrylamide. We thus varied AHA concentration to obtain different levels of ECM tethering (Fig. 1b), estimating that the surface density of ECM anchorage points approximately triples (see the “Methods” section) when AHA concentration is increased from 16 mM (low AHA) to 48 mM (mid AHA) and 80 mM (high AHA) (Fig. 1b). We adapted the ratio of acrylamide to bis-acrylamide to ensure a constant stiffness over the range of AHA concentrations (Fig. 1c).

It is not immediately clear how control over ECM tethering might translate to how cells sense the ECM. One possibility is that ECM protein coverage varies with increasing density of tethering points. However, this is unlikely because we are working with saturating ECM concentrations. As expected, immunofluorescence confirmed that protein coverage levels were similar on soft and stiff substrates over a range of AHA concentration (Fig. 1d and Supplementary Fig. 1a). The other potentially important parameter is how strongly ECM proteins are tethered to the substrate, which varies not only with substrate type but also potentially with density of tethering points. In order to measure ECM tethering strength, we used AFM cantilevers coated with an anti-fibronectin antibody. On both soft and stiff StemBond hydrogels, we detected significant binding events (Supplementary Fig. 1b–e). We did not, however, measure significant binding events on sulfo-SANPAH functionalised stiff hydrogels, primarily because the rupture length was very short on these gels (Supplementary Fig. 1e). The short rupture length on stiff sulfo-SANPAH substrates, which have relatively high ECM tethering density, likely indicates a weak tethering of ECM to the substrate. On soft sulfo-SANPAH substrates, the significant binding events had longer rupture lengths but significantly smaller rupture forces than on soft StemBond substrates (Fig. 1e and Supplementary Fig. 1b–e), also indicative of weaker ECM tethering to the substrate. Plateauing rupture forces on high AHA hydrogels suggest saturation of ECM tethering points. Notably, the rupture forces are very similar for both soft and stiff StemBond hydrogels (Supplementary Fig. 1b and e). Our analysis strongly suggests that the increasing concentration of AHA does not significantly affect ECM protein coverage, but, in line with previous studies^29^, that it has a potent effect on ECM tethering strength, and thus potentially on ECM stability.

To determine the stability of the ECM layer on cell-seeded hydrogels, we plated mouse embryonic fibroblasts (MEFs), which are a strongly adherent cell type that secrete and remodel ECM, on both StemBond and sulfo-SANPAH hydrogels coated with the same concentration of ECM. We observed that, on StemBond, the ECM layer remained on the surface even after several days; however, significantly, the ECM layer on sulfo-SANPAH treated hydrogels was depleted. This observation strongly suggests that the ECM is more stable on StemBond hydrogels (Fig. 1f), and this stability correlates with the tethering strength. Thus, controlling co-factor concentration on StemBond substrates allowed stable, reproducible, and tuneable ECM tethering, on both soft and stiff hydrogels.

### StemBond hydrogels promote strong pluripotent stem cell attachment

We then tested whether strong ECM tethering would improve stem cell attachment. We first seeded mESCs on standard and StemBond PAAm hydrogels. We compared hydrogels of different pore size (varying acrylamide:bis-acrylamide ratio A:B) and sulfo-SANPAH concentrations. We found that prolonged cell attachment on fibronectin-coated StemBond hydrogels was significantly higher than on standard PAAm hydrogels functionalised with sulfo-SANPAH (Supplementary Fig. 2a, b). Notably, after a few days of culture on sulfo-SANPAH functionalised hydrogels, independent of concentration, there were clear floating or barely attached colonies, and these hydrogels ultimately yielded as few attached colonies as the unfunctionalised hydrogels. In contrast, on StemBond hydrogels we observed many large colonies irrespective of A:B ratio (Supplementary Fig. 2a, b).

**Fig. 2:**
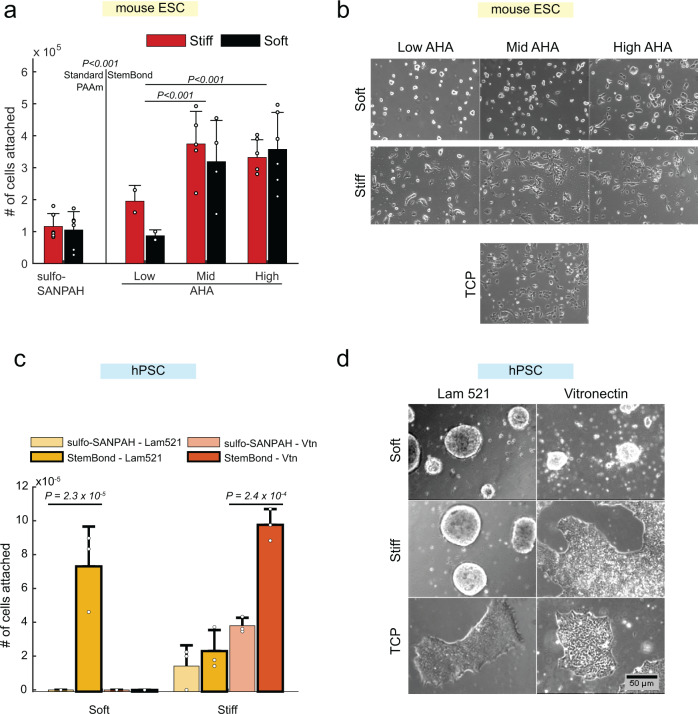
StemBond improves cell attachment to soft hydrogels. **a** mESC attachment on sulfo-SANPAH functionalised standard PAAm hydrogels (left) and StemBond hydrogels (right). Cells were counted after 48 h in serum+LIF (*n* = 2–6 independent samples). *P*-values from ANOVA with Tukey–Kramer’s multiple comparison test comparing Low AHA to Mid AHA and High AHA, and sulfo–SANPAH hydrogels to StemBond hydrogels globally. Stiffness was not a significant factor (*p* = 0.58). Bars show mean ± standard deviation, points show the value independent samples. **b** Representative brightfield images of cells after 24 h in serum + LIF on tissue culture plastic (TCP), stiff and soft StemBond hydrogels with different adhesiveness (AHA concentrations). Scale bar: 100 µm. **c** hPSC attachment on soft and stiff sulfo-SANPAH functionalised standard PAAm hydrogels and high AHA StemBond hydrogels, with laminin 521 or vitronectin coating. Cells were counted after 4 days in E8 media (*n* = 3 independent experiments). *P*-values were computed using a one-way ANOVA with Tukey–Kramer’s multiple comparison test, comparing sulfo-SANPAH functionalised to StemBond hydrogels for each stiffness and ECM protein. On soft substrates, only Laminin on StemBond yielded enough cells, other conditions yielded very little cells, below the range of the cell counter. Error bars show standard deviation, points show the value independent repeats. **d** Representative brightfield images of hPSC colonies attached to laminin 521 and vitronectin coated StemBond hydrogels at day 4 of seeding (day 2 for TCP). Cells were grown in E8 media with ROCK inhibitor for the first 24 h. Scale bar: 50 µm. Source data are provided as a Source Data file.

We further tested whether different levels of AHA would impact cell attachment over the course of 48 h. We found that cell attachment was long-lasting on mid and high AHA hydrogels, irrespective of stiffness, and weaker on the low AHA hydrogels only (Fig. 2a), which had similar attachment numbers to standard PAAm hydrogels. Immunostaining confirmed that markers of focal adhesions, integrin β1 and phospho-paxillin, were present on soft and stiff hydrogels (Supplementary Fig. 2c). Actin stress fibres were also observed on tissue culture plastic (TCP) and stiff hydrogels, but actin was mostly cortical on soft substrates (Supplementary Fig. 2c). Furthermore, cell attachment and viability were comparable to TCP (Supplementary Fig. 2d, e). Proliferation rates assessed by EdU incorporation were approximately the same for all substrates (Supplementary Fig. 2f). Thus, StemBond hydrogels, in contrast to sulfo-SANPAH functionalised hydrogels, provide robust conditions for prolonged culture of mESCs, even on soft substrates.

Morphologically, colonies on soft StemBond hydrogels were rounder, resembling naïve mESCs in 2i culture; on stiff StemBond hydrogels, colonies were flat and spread out, similar to mESCs on TCP (Fig. 2b). ECM tethering density did not dramatically impact morphology, though we observed that colonies were systematically slightly rounder on, particularly, the low and mid AHA soft substrates (Fig. 2b). Similar morphological differences between soft and stiff substrates were found on gelatin, laminin and collagen-coated StemBond hydrogels, although mESCs did not flatten as much on collagen (Supplementary Fig. 3a). We also compared StemBond hydrogels to other types of soft substrates. On soft PDMS substrates, cells spread out to a level similar TCP (Supplementary Fig. 3b). StemBond hydrogels are therefore unique in providing a substrate viable for mESC attachment, and recapitulating naïve state morphology.

To show that StemBond hydrogels are compatible with multiple pluripotent stem cell types, we also established hPSC culture on StemBond hydrogels. We here compared laminin 521 (Lam 521) and vitronectin (Vtn) coating, both routinely used for hPSC culture, on either high AHA StemBond or sulfo-SANPAH functionalised hydrogels. Similar to our examination of mESCs, we found that sulfo-SANPAH does not provide a stable culture environment for hPSCs and leads to rapid colony detachment (Fig. 2c). In contrast, StemBond hydrogels allowed robust colony attachment and growth. Interestingly, we observed that only some combinations of stiffness and ECM show optimal colony attachment. Laminin 521 is highly supportive of colony growth on soft substrates, with colonies appearing round on both soft and stiff StemBond hydrogels, while vitronectin supports colony growth only on stiff substrates (Fig. 2c, d). These results suggest that the interaction between ECM protein and stiffness regulate cell attachment and cell growth.

### Soft substrates support naïve pluripotency

**Fig. 3:**
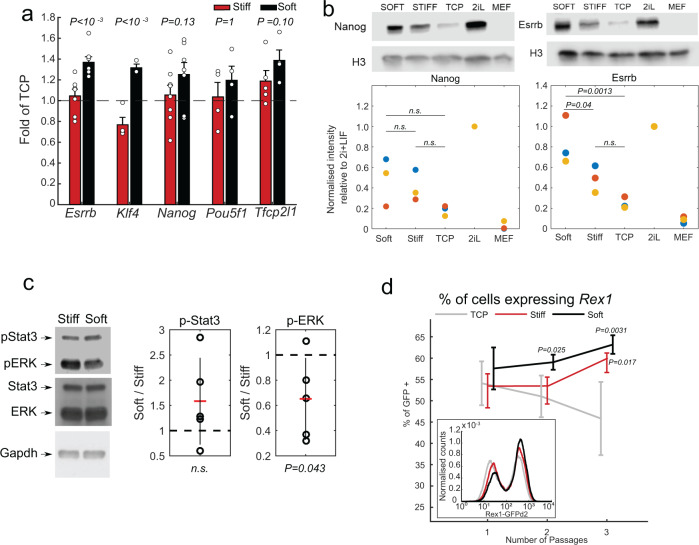
StemBond substrates support pluripotency. **a** Mean and standard deviation of mRNA expression of selected pluripotency genes on soft and stiff (mid AHA) substrates normalised to TCP. Cells were seeded on the substrates for 24 h in serum + LIF. The *P*-values show the significant differences in expression between soft and stiff substrates (one-way ANOVA). Expression was averaged over *n* = 4–6 independent experiments, points show the value of independent repeats. **b** Quantification of NANOG (left) and ESRRB (right) on different substrates from Western blots. Scatter plots show normalised band intensity from three independent experiments. Cells were cultured for 48 h in serum + LIF on fibronectin-coated TCP, soft and stiff hydrogels. Intensity were normalised to protein levels in 2i+LIF (2iL). Mouse embryonic fibroblasts (MEFs) were used as negative controls. *P*-values are from one-way ANOVA test. **c** (Left) Western blots for phospho-STAT3, phospho-ERK, total STAT3 and total ERK from cells grown on soft and stiff gels for 24 h in serum+LIF. GAPDH was used as loading control. (Right) Mean ± std of the ratio of intensity of soft/stiff after normalisation to GAPDH (*n* = 5 independent experiments). *P*-value computed using a one-way ANOVA. **d** Average percentage of reporter line Rex1 positive cells determined from flow cytometry histograms. Cells were cultured in serum + LIF on TCP (grey), and on soft (black) and stiff (red) high AHA substrates (*n* = 4 independent experiments). *P*-values from two-way ANOVA are indicated. Bars show mean ± standard deviation. Inset: Corresponding example of flow cytometry profiles. For each condition, histograms of three replicate samples were averaged and smoothed. Gating strategy show in Supplementary Fig. 4a. Source data are provided as a Source Data file.

Having established that StemBond hydrogels provide robust cell attachment, we next asked if they also support maintenance of pluripotency in mESCs and hPSCs. To do this, we first examined several canonical naïve transcription factors of mESCs, finding that the expression of those factors is systematically higher on soft substrates compared to TCP and stiff substrates. We found, for example, a significant increase in the expression of *Esrrb* in comparison to stiff substrates (Fig. 3a). At the protein level, ESRRB was also significantly more expressed on soft substrates compared to stiff hydrogels and TCP, though we did not find such a dramatic difference in NANOG (Fig. 3b). In addition, we probed the activity of STAT3 and ERK, which are known regulators of mESC cell state, on soft substrates compared to stiff substrates in serum + LIF conditions. STAT3 activity was higher but not significantly different, while ERK activity was significantly lower, suggesting that soft substrates stabilise naïve pluripotency by reducing ERK-driven differentiation and potentially by increasing self-renewal signals such as STAT3 (Fig. 3c). Finally, comparing gene expression on soft StemBond hydrogels to soft PDMS substrates on both fibronectin- and laminin-coated substrates, we showed that StemBond hydrogels lead to higher expression of naïve factors *Esrrb, Tfcp2l1* and *Klf4* and lower expression of formative marker *Fgf5* than PDMS (Supplementary Fig. 3c). Together, these results confirm that StemBond hydrogels fully support the maintenance of naïve pluripotency in mESCs, with the expression of key pluripotency factors being further increased on soft substrates compared to both stiffer substrates, and other hydrogel substrates such as PDMS.

To determine more closely the population response on StemBond hydrogels, we used a destabilised GFP reporter line for *Rex1* (*Zfp42*), a high-fidelity reporter for naïve pluripotency^31^, and measured fluorescence intensity over several passages. While a bimodal distribution typical of serum + LIF conditions was observed in all conditions, we found that soft substrates significantly increased the proportion of *Rex1*-positive cells without shifting the peak fluorescence of the naïve cells (Fig. 3d). Additionally, this proportion increased over time in culture on both soft and stiff hydrogels, but not on TCP (Fig. 3d). These data suggest that in serum + LIF conditions StemBond hydrogels—particularly the soft substrates—progressively improve the homogeneity of the mESC population.

In contrast to serum + LIF, the 2i conditions defined by the use of CHIRON (Gsk3β inhibitor) and PD03 (MEK inhibitor) create a homogeneous naïve population from which mESCs can be differentiated into multiple lineages. In 2i conditions, mESCs were homogeneously naïve regardless of substrate (Supplementary Fig. 4a) and mESCs exited from naïve pluripotency on all substrates (Supplementary Fig. 4a).

Given that exit from naïve pluripotency was unaffected by substrate type, we then asked if multilineage specification of mESCs was similarly unaffected. For this, we differentiated mESCs starting from 2i conditions into neuroectoderm and mesoendoderm^32^. When prompted to differentiate into neuroectoderm, cells upregulated *Pax3, Pax6* and *Sox1*, markers of early neuroectoderm lineage commitment on both soft and stiff hydrogels (Supplementary Fig. 4b, c) within 4 days. When prompted to differentiate towards mesoendoderm, cells showed normal upregulation of lineage markers *Eomes*, *Foxa2* and *T*/*Brachyury* within 4 days (Supplementary Fig. 4b, c). Overall, we conclude that, though naïve pluripotency is stabilised on StemBond hydrogels, mESCs are able to differentiate on these substrates, and that both exit from naïve pluripotency and lineage priming proceed normally regardless of substrate condition.

We next verified that StemBond hydrogels support pluripotency in hPSCs. General pluripotency markers (*Pou5f1* and *Nanog*) were similar in hPSCs on TCP and StemBond hydrogels coated with either laminin and vitronectin, despite the weaker attachment on soft vitronectin substrates (Supplementary Fig. 3d). Furthermore, although the ECM protein was shown to make a difference on cell attachment and cell morphology (Fig. 2d), this did not affect pluripotency gene expression in hPSCs. Only expression of *Nanog* on vitronectin-coated TCP was lower than in other conditions. In naïve hPSCs, gene expression was overall comparable for the naïve markers *Klf4*, *Klf17*, and *Tfcp2l1* on TCP and StemBond hydrogels for HNES1 and cR-H9 cells (Supplementary Fig. 3e). Thus, we conclude that StemBond hydrogels are compatible with culture and propagation of hPSCs without the requirements for feeder layers or Matrigel.

### Pluripotent stem cell self-renewal in minimal media conditions

**Fig. 4:**
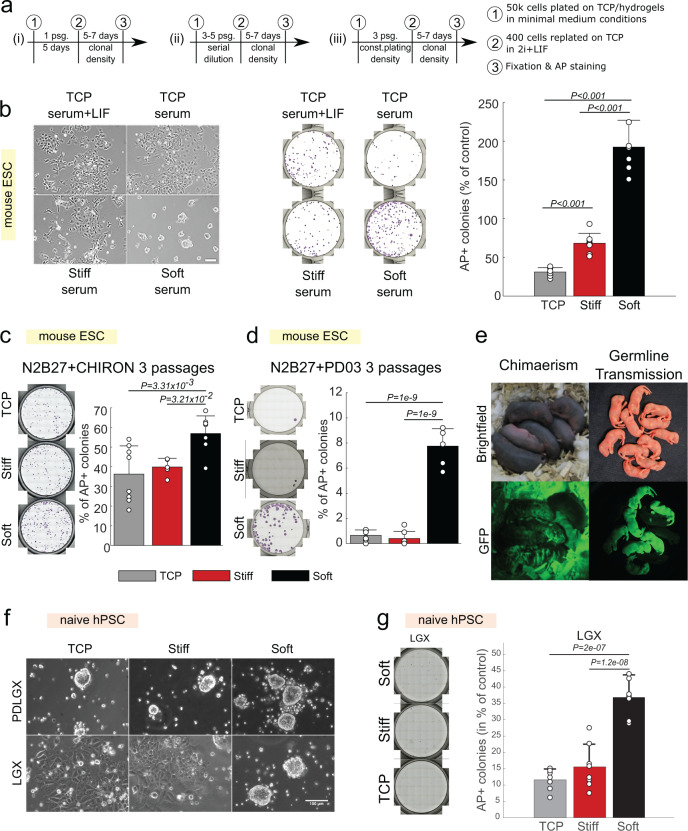
Soft substrates improve self-renewal in minimal conditions. **a** Procedure for clonogenicity assays. Cells were seeded on different substrates (i) for 5 days or (ii) and (iii) passaged every 2–3 days. Passaging (psg.) was done (ii) by serial dilution from 1:3 to 1:4 or (iii) by keeping a constant plating density of 50,000 cells per substrate. Cells were then replated at clonal density (100 cells/cm²) on gelatin-coated TCP in 2i+LIF to allow self-renewing cells to form colonies. Finally, colonies were fixed and stained for alkaline phosphatase (AP). **b** Clonogenicity assay of mESCs after 5 days in serum following protocol (i). (Left) Brightfield images of mESCs after 5 days. Scale bar: 100 µm. (Centre) Snapshot of AP stainings. (Right) Quantification of the number of AP+ colonies in % of control conditions (serum + LIF on TCP) (*n* = 8 independent samples). **c** Clonogenicity assay after mESCs were cultured for three passages in N2B27 + CHIRON following protocol (iii). (Left) Snapshots of AP stainings. (Right) Quantification of the number of AP + colonies in % of plated cells (*n* = 5–8 independent samples). **d** Clonogenicity assay after mESCs were cultured for three passages in N2B27 + PD03 following protocol (iii). Cells were from a C57BL/6-Agouti background. (Left) Snapshots of AP stainings. (Right) Quantification of the number of AP+ colonies in % of plated cells (*n* = 8 independent samples). **e** First (left) and second (right) generation chimaeric mice obtained after the injection into blastocysts of GFP-transfected mESCs cultured on soft substrates in N2B27 + PD03 for three passages following protocol (ii). The bedding autofluorescence contributes to background in the bottom-left image, but clear GFP+ regions are visible on the mice. **f** Brightfield images of naïve hPSCs in control media “PDLGX” and in media without PD03, “LGX”. Scale bar: 100 µm. **g** Clonogenecity assay of naïve hPSCs in LGX. (Left) Snapshot of AP-stainings. (Right) Quantification of number of AP+ colonies in % of control conditions “PDLGX”. The replating efficiency was low with only 11% recovered colonies in PDLGX conditions (*n* = 8 independent samples). In all panels above, error bars show standard deviation, points show the values of independent repeats, and *P*-values were computed using a one-way ANOVA with Tukey–Kramer’s multiple comparison test. Source data are provided as a Source Data file.

So far, we have established that StemBond hydrogels support mESC and hPSC self-renewal. In mESCs, the purity of the naïve population increased in serum + LIF conditions, although not to the level observed in 2i conditions. Nevertheless, the small molecule inhibitors and LIF are potent self-renewing factors that could mask any difference emerging from substrate conditions. In order to challenge the system, we then asked if the above-mentioned factors are necessary for mESC self-renewal on StemBond hydrogels by conducting clonogenicity assays in minimal media conditions. In these assays, we removed one or several self-renewing factor(s) for a defined period of time, then replated the cells at very low densities in 2i + LIF medium for 5 days to select for cells that retained their ability to self-renew in naïve conditions. We then counted the number of resulting naïve pluripotent colonies (Fig. 4a(i)). First, we removed LIF (a positive effector of self-renewal) from serum + LIF medium for 5 days on TCP, stiff and soft StemBond substrates. On TCP, removal of LIF led to drastic loss of self-renewal. On stiff substrates, there was a modest loss of naïve pluripotency upon removal of LIF. On soft substrates, however, significantly more cells maintained naïve pluripotency than on stiff substrates and TCP (Fig. 4b). Surprisingly, twice as many cells maintained naïve pluripotency on soft substrates without LIF when compared to the control of undifferentiated cells grown on TCP in serum + LIF. These results suggest that in serum only conditions, StemBond hydrogels at least partially compensate for LIF to sustain self-renewal of mESCs.

We then tested whether soft substrates could compensate for one of the two otherwise essential inhibitors, CHIRON or PD03, and would allow the propagation of mESCs for multiple passages in minimal media conditions. For this, we cultured cells on different substrates for five passages in N2B27 + CHIRON by serial dilution, before replating them at very low density in 2i + LIF to assess how many cells remained in a naïve pluripotent state (Fig. 4a(i)). We found that significantly more cells gave rise to naïve colonies from soft substrates than from stiff substrates (Supplementary Fig. 5a, b). Moreover, cell survival was also significantly higher on soft substrates (Supplementary Fig. 5c). Since the higher survival could be a confounding factor because it influences plating density in a serial dilution experiment, we repeated the experiment keeping constant cell plating density at each passage (Fig. 4a(iii)). After three passages of constant plating density, there was low cell survival on stiff substrates, but survival was still high on soft substrates. Furthermore, we found in these conditions that there were significantly more naïve pluripotent cells on soft substrates than on stiff substrates and TCP (Fig. 4c).

In N2B27 + PD03, cell survival was too low to perform the serial dilution clonogenicity assay. We therefore kept a constant cell plating density over multiple passages (Fig. 4a-iii). This allowed propagation for up to three passages on soft and stiff substrates only, with no cells surviving past passage 2 on TCP (Supplementary Fig. 5d), in line with previous studies^17^. Survival did gradually decrease on soft and stiff substrates, with no significant differences in cell numbers, and <10% of cells giving rise to naïve colonies on both substrates (Supplementary Fig. 5e, f). Because sensitivity to MEK/ERK inhibition (but not to GSK3β inhibition) has been shown to depend on cell line^17^, we repeated this assay with a different cell line, from a C57BL/6-Agouti background. In this case, we found a greater effect of the substrate stiffness on the number of naïve pluripotent colonies obtained with ~9% on soft substrates but only ~1% on stiff substrates and TCP (Fig. 4d). To ultimately confirm that the cells cultured in N2B27 + PD03 on soft hydrogels were still naïve pluripotent, we injected GFP-targeted mESCs into mouse blastocysts to test their ability to contribute to the germ layers and the germline. We obtained five viable chimaeras from the first injection and seven GFP-expressing pups from the next generation, demonstrating germline transmission (Fig. 4e and Supplementary Table 1). To our knowledge, this is the longest that mESCs have ever been successfully maintained in this highly challenging condition without genetic manipulation. We conclude that soft substrates more robustly provide a microenvironment supportive of self-renewal in N2B27 + PD03.

MEK inhibition is also used to maintain naïve hPSCs (HNES1)^33,34^. We thus asked whether soft substrates would also support self-renewal of HNES1 cells in absence of MEK inhibition, as is the case for mESCs. We found that, after 3 days without MEK inhibition, ERK activity was higher on TCP compared to soft but also compared to stiff substrates (Supplementary Fig. 5g), suggesting that ECM–substrate attachment could impact ERK activation. We found a relative higher expression in naïve markers *Klf4* and *Klf17* after 4 days without MEK inhibition on soft substrates compared to TCP and stiff hydrogels (Supplementary Fig. 5h). We further cultured HNES1 in control conditions or without PD03 for 5–6 days before replating them at clonal density and staining for naïve colonies (as in Fig. 4a(i)). The number of growing replated colonies in control conditions did not vary significantly between substrates, but in absence of PD03, soft StemBond substrates yielded 2.5 times more colonies than stiff substrates and 3 times more than TCP (Fig. 4f), mirroring the above results with mESCs. Taken together, we conclude that both stiffness and substrate type impact pathway activity, ultimately resulting in increased self-renewal of mESCs and hPSCs on soft StemBond hydrogels cell in minimal media.

### Soft StemBond hydrogels promote acquisition of naïve pluripotency

**Fig. 5:**
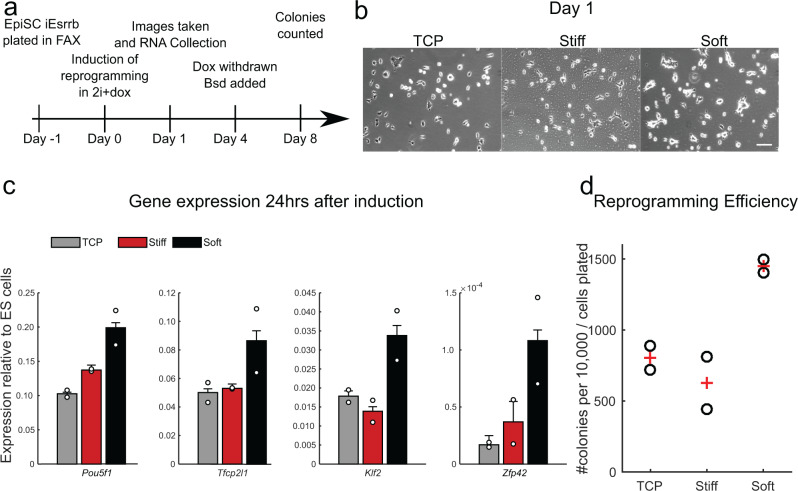
mEpiSC reprogramming is boosted on soft substrates. **a** Schematic of reprogramming procedure. EpiSCs were plated in their maintenance Fgf2 + ActivinA + XAV (FAX) medium. *iEsrrb*-driven reprogramming was induced by switching to 2i+doxycycline (dox) the next day. After 4 days of reprogramming, Dox was withdrawn and blasticidin (bsd) was added to select for naïve colonies with an active *Rex1* promoter. Blasticidin-resistant iPSC colonies were counted on day 8. **b** Phase contrast images 24 h after induction of *iEsrrb* EpiSC reprogramming in 2i+dox on TCP, stiff and soft substrates. Scale bar, 100 µm. **c** Gene expression profile 24 h after induction of *iEsrrb* EpiSC reprogramming in 2i+dox. Expression is presented relative to *Gapdh* then normalised to 2i+LIF mESC levels. Circles represent individual data points, bars show average (*n* = 2). **d** Number of iPSC colonies counted on day 8 on TCP, stiff and soft substrates, as a measure for reprogramming efficiency, circles show individual data points, ‘+’ show average (*n* = 2 samples). See also Supplementary Fig. 6. In all panels, error bars show standard deviation. Source data are provided as a Source Data file.

To illustrate how StemBond substrates could be used across different stem cell types, we turned to primed mouse epiblast stem cells (mEpiSC), which are known to require stronger cell adhesion than mESCs. mEpiSC can be reprogrammed to induced naïve pluripotent stem cells (iPSC) by the forced expression of a single naïve transcription factor together with signalling cues^35–37^. We here hypothesised that soft StemBond hydrogels could improve the induction of naïve pluripotency. We employed doxycycline-inducible *Esrrb* (iEsrrb) as an efficient driver of reprogramming in EpiSCs^38–40^ with a *Rex1::dGFP-IRES-bsd* reporter for the naïve identity^31^. mEpiSCs did not stably adhere to sulfo-SANPAH treated substrates and only weakly to mid AHA StemBond hydrogels, but they adhered well to high AHA StemBond hydrogels, which we thus used for reprogramming assays. We plated mEpiSCs on plastic, stiff and soft substrates for 24 h before inducing reprogramming with 2i+doxycycline and then assessed the efficiency of the process at different time points (Fig. 5a). After 24 h, cells were more clustered together on soft substrates than on TCP and stiff hydrogels (Fig. 5b). Both early markers of reprogramming, *Tfcp2l1* & *Klf2* and the late marker *Zfp42* (*Rex1*) showed a twofold increase in gene expression on soft substrates. The general pluripotency marker *Pou5f1* (Oct4), which transiently drops when reprogramming is triggered, was more effectively maintained on soft substrates (Fig. 5c). These results suggested a boost in the naïve pluripotency network during the reprogramming process. Correspondingly, we found a higher end-point efficiency of reprogramming on soft substrates, with twice as many naïve colonies on soft substrates at day 8 (Fig. 5d). Naïve genes were expressed at similar levels in resultant iPSCs demonstrating full reprogramming (Supplementary Fig. 6). Therefore, soft StemBond hydrogels not only support mESC self-renewal but also promote specification of naïve pluripotency during reprogramming.

### Substrate stiffness induces genome-wide transcriptomic changes

**Fig. 6:**
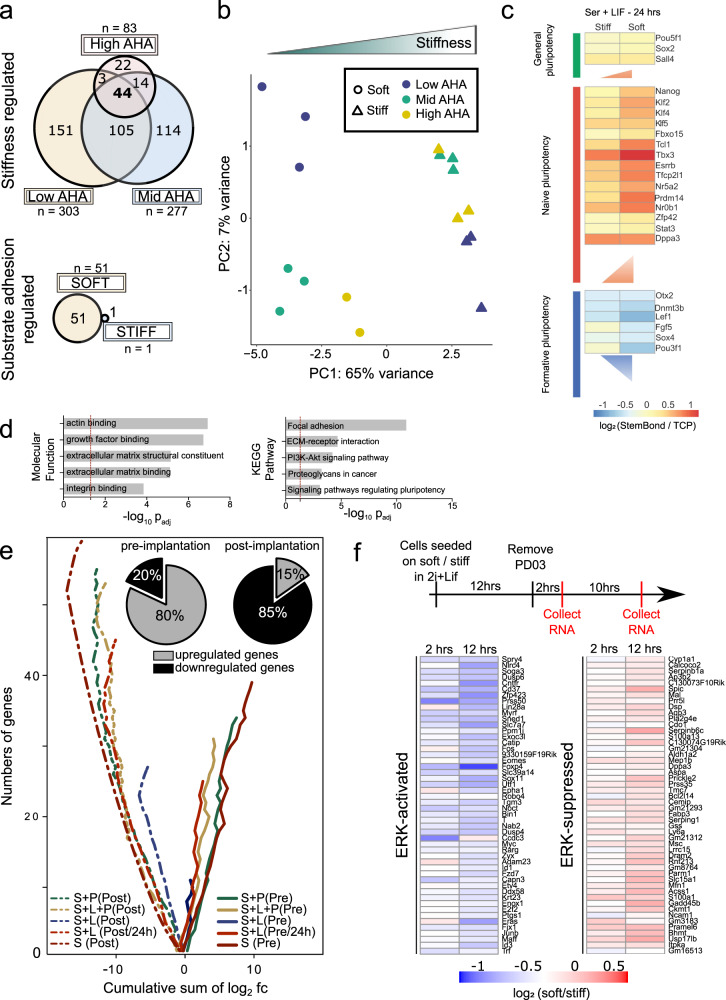
Substrate stiffness modulates the transcriptome of mESCs. **a** Venn diagrams indicating the number of differentially expressed genes (*p*_adj_ < 0.05) in pair-wise comparisons. (Top) Number of genes regulated by substrate stiffness for low (*n* = 303), mid (*n* = 277) or high (*n* = 83) ECM tethering density. (Bottom) Genes regulated by ECM tethering on soft (51) and stiff (1) substrates. **b** Principal component analysis (PCA) computed on differentially expressed genes across all conditions (one point = one sample). Conditions are indicated by markers (stiffness) and colour-code (AHA concentration). **c** Heatmap of log_2_fold change of expression for a selection of general pluripotency, naïve pluripotency and formative genes. All media conditions are compared to TCP control (log_2_(hydrogel/TCP)). The vertical side of each triangle is proportional to the average log_2_fc(soft/stiff) over all genes of a cluster. **d** Molecular functions and KEGG pathways that are enriched in the systematically modulated genes (*n* = 219). Dotted red lines represent the significant threshold (*p*_adj_ < 0.05). See also Supplementary Fig. 8d. **e** Cumulative sum of log_2_fc for all genes (abs(log_2_fc) > 0.2) belonging to pre-implantation (solid lines) or post-implantation (dotted lines) clusters. The diverging lines of the pre- and post-implantation genes indicate some systematic and opposing effect of substrate stiffness on those clusters (otherwise one would expect the solid and dotted lines would converge around zero cumulative log_2_fc). Pie charts show the average percentage of significantly up/downregulated genes over all conditions (percentages for each condition given in Supplementary Fig. 8F). S: serum; S + L: serum+LIF; L: LIF; P: PD03; Pre:pre-implantation; Post: post-implantation. All time points were 48 h, except for “S + L 24 h”. log_2_fc = log_2_ fold change = log_2_(soft/stiff). **f** (Top) Design of experiments for RNA-sequencing indicating the timing of the different steps and PD03 removal. Experiments performed in triplicate. (Bottom) Heatmaps of log_2_ fold change in expression between soft and stiff substrates after PD03 removal. (Left) Top 50 activated genes and (right) Top 50 suppressed genes. The genes listed are those that are co-regulated after inhibitor removal on both soft and stiff and show the largest fold change on stiff substrates when compared to control in 2i+LIF.

To demonstrate potential applications of StemBond hydrogels, we used them to gain deeper insight into the potential synergies between adhesion, stiffness, and regulation of naïve pluripotency. We here examined the genome-wide transcriptional changes triggered by different substrate conditions in mESCs. We first determined the impact of substrate stiffness and ECM tethering on mESC gene expression by performing RNA sequencing in serum + LIF conditions (Supplementary Dataset 1). We found that substrate stiffness had a greater influence on gene expression than ECM tethering strength, with 452 differentially expressed genes due to changes in stiffness along with greater overall fold changes in gene expression, against 52 differentially expressed genes due to ECM tethering. The genes that were differentially expressed due for different ECM tethering strengths were primarily observed on soft substrates (Fig. 6a). Principal component analysis (PCA) confirmed that samples primarily cluster by substrate stiffness rather than by substrate adhesiveness (Fig. 6b). Among the genes differentially regulated by substrate stiffness are naïve pluripotency factors, as already observed by qPCR (Fig. 3a). In contrast, the same markers showed no significant differences in gene expression between our low AHA and high AHA substrates (Supplementary Fig. 7a), confirming that ECM tethering is not a significant factor in the regulation of naïve pluripotency. Gene ontology analysis of the differentially expressed genes revealed enrichment for stem cell population maintenance and transcription processes in genes upregulated on soft substrates, whereas cell adhesion and migration as well as some differentiation pathways were enriched in downregulated genes (Supplementary Fig. 7b). We conclude that while sufficient substrate adhesiveness is essential for the prolonged attachment of mESCs, substrate stiffness is the key mechanical property impacting the regulation of naïve pluripotency in mESCs.

To further assess if the observed stiffness sensitivity of naïve mESCs was dependent on LIF or ERK signalling, two signals known to be important for maintenance and differentiation of mESCs respectively, we again used RNA sequencing of mESCs in serum with or without LIF and/or without the MEK/ERK inhibitor, PD03, now focussing on a single adhesiveness condition (mid AHA). We found that, in all media conditions, substrate stiffness had a significant impact on gene expression, with over 210 genes being modulated independently of the presence of LIF or PD03 (in bold in Supplementary Fig. 7c). We first examined the expression of naïve and of recently defined formative genes^31,41^, the latter being indicative of cells having exited naïve pluripotency. In detail, we found that the expression of most naïve pluripotency factors, but not of general pluripotency factors, was significantly increased on soft compared to stiff hydrogels and to TCP, whereas the expression of formative genes was significantly decreased on soft substrates (Fig. 6c, Supplementary Fig. 7d–f and Supplementary Dataset 2). Notably, these changes were more pronounced after 24 h in culture as opposed to 48 h (Supplementary Fig. 7d), suggesting that phenotypic differences begin to be masked at high cell density. The changes in expression levels were also observed across different media conditions (Supplementary Fig. 7d, e), and most pronounced in the absence of LIF and PD03. Consistent with these results, PCA revealed that samples separate based on stiffness, more so in absence of LIF (Supplementary Fig. 8a). Taken together, these results suggest that biological processes active in primed cells could be specifically stiffness-sensitive, and that this stiffness-sensitivity is mediated by interactions between substrate stiffness and the LIF/STAT3 and MEK/ERK pathways, which respectively drive naïve self-renewal^18^ and early differentiation^42,43^.

Gene ontology analysis on the genes differentially regulated by substrate stiffness (in serum ± LIF ± PD03) showed that beyond naïve pluripotency and formative genes, functions related to the cytoskeleton and the extracellular matrix were also differentially enriched, as well as signalling pathways involved in self-renewal and pluripotency (Fig. 6d). The transcription of key mechanosensing structures, such as focal adhesions, cell–cell junctions, and the cytoskeleton, was also differentially regulated on soft and stiff substrates in all conditions (Supplementary Fig. 8b–d). When further scrutinising the enriched pathways, we found that, on average, 74% of differentially regulated components known to be involved in epithelialisation, cell adhesion and cytoskeleton reorganisation were downregulated on soft substrates in serum conditions (Supplementary Fig. 8e, Supplementary Dataset 2).

Since epithelialisation is essential to epiblast progression in vivo ^44^, these results prompted us to ask if the observed stiffness-dependent gene expression might have significance in the context of embryonic development. To do this, we computed the log_2_ fold change of the differentially expressed genes that were associated either to the pre- or post-implantation epiblast (previously characterised by sequencing early embryos^45^) and found that among pre-implantation genes, 80% were upregulated on soft substrates, while among the post-implantation genes, 85% were downregulated on soft substrates (Fig. 6e, Supplementary Fig. 8f, and Supplementary Dataset 2). The trend was similar across all media conditions. Thus, mESCs cultured on soft and stiff substrates recapitulated key transcriptional differences between pre-implantation epiblast and post-implantation epiblast, respectively.

Substrate stiffness sensing therefore not only impacts the organisation of the cytoskeleton (as shown in Supplementary Fig. 2c) but also feeds back onto the transcriptional control of cytoskeleton-driven processes. Altogether, our RNA sequencing analysis indicates that biological processes occurring upon post-implantation, among which is epithelialisation, are sensitive to substrate stiffness. We speculate that soft substrates reduce the background occurrences of epithelialisation and cytoskeleton remodelling in serum-rich media, thereby promoting self-renewal in minimal conditions, as we observed above (Fig. 4). Stiff substrates on the other hand promote background differentiation in serum-rich media.

The substrate dependence of ERK activity, a driver of early differentiation in mESCs, was already indicated by protein assays after 24 h in serum + LIF (Fig. 3c). To probe this at the transcriptional level, we identified ERK targets by carrying out RNA-sequencing at early time-points (2 and 12 h) after removing PD03 from 2i + LIF media (Fig. 6f and Supplementary Dataset 3). Note that CHIRON + LIF media is sufficient to maintain naïve pluripotency, so this assay tests specifically for the effects of ERK signalling induction in mESCs independent of changes in pluripotent state. Known ERK targets such as Dual-Serine Phosphatases (*Dusps*) and Immediate-Early Genes (*Fos, Egr1, Jun*) were all activated after 2 h on soft and stiff substrates, confirming that PD03 withdrawal led to ERK activation in all conditions. We observed a systematic effect of substrate stiffness on the magnitude of ERK-target regulation (Fig. 6f). A comparison of the expression of all co-regulated genes (i.e. either activated or suppressed by ERK on both substrates) revealed that nearly 90% of ERK-activated genes were downregulated on soft substrates, whereas 85% of ERK-suppressed genes were upregulated on soft substrates compared to stiff ones (Supplementary Fig. 9b). This was already evident 2 h after removing PD03, and significantly more so at 12 h. The absolute fold change was significantly larger for ERK-regulated genes compared to non-target genes (Supplementary Fig. 9c). Additionally, we computed log_2_ fold changes of ERK target genes in different media conditions and on different adhesiveness (data sets of Fig. 6a, b and c–e) and found similar trends, whereby ERK-suppressed genes were higher on soft substrates, but ERK-activated genes were lower. Other variables did not have a significant impact (Supplementary Fig. 9d).

In order to see if GSK3/β-catenin activity, which also regulates pluripotency, is similarly affected by substrate stiffness, we performed the same analysis removing CHIRON from 2i + LIF. Both canonical and non-canonical Wnt pathway genes were suppressed on soft and stiff substrates when CHIRON is removed. However, we observed no dependence on substrate-stiffness, even after 12 h (Supplementary Fig. 10 and Supplementary Dataset 4). This suggests that the GSK3/β-catenin pathway is not sensitive to substrate stiffness in mESCs. Taken together, our results suggest that substrate stiffness significantly impacts the activation and transcriptional activity of the ERK pathway, pointing to ERK mechanosensitivity as a primary mechanism for the optimised maintenance of self-renewal on soft substrates.

## Discussion

In this study, we characterised a range of two-dimensional substrates for stem cell culture that allow robust cell–ECM adhesion as well as self-renewal and differentiation. A handful of other studies have incorporated groups that can selectively and specifically bind ECM proteins directly into the hydrogel precursor solution^5,29,46^ or on the surface of a polymerised gel^47^. Here, we adopted a method (i.e. incorporating AHA co-factor) that allowed us to vary the strength of ECM tethering, and adapt it for culture of various stem cell types. We found that the resulting StemBond hydrogels perform markedly better than existing standards and commercially available substrates in ensuring equally robust stem cell–ECM attachment at both extremes of the stiffness range. Our results suggest that unstable ECM–substrate crosslinking, not pore size, is responsible for poor pluripotent stem cell attachment with sulfo-SANPAH functionalisation. Notably, hPSCs detached immediately after removal of ROCK inhibition on sulfo-SANPAH hydrogels but not on StemBond hydrogels, indicating, at least for some cells, a critical requirement for ECM stability under cell-generated traction forces. By engineering a stable ECM–substrate attachment, StemBond hydrogels thus allow more systematic interrogation of stem cell mechanosensitivity. It remains to be determined whether ECM stability is also important in 3D systems, in which ECM degradability was shown to be a critical factor in differentiation of cardiomyocytes and mesenchymal stromal cells^48,49^. In the future, StemBond hydrogels will allow to further disentangle the connection between stiffness, ECM composition, tethering strength, and ECM stability.

Our results here showed that not only substrate stiffness, but adhesion strength and ECM protein type is important for pluripotent stem cell culture. mESCs attached best on mid-to-high AHA concentrations, although their self-renewal was unaffected by AHA concentration. hPSCs and mEpiSC, both in a primed pluripotency state, attached better on high AHA substrates, possibly reflecting that primed cells have greater requirements for strong ECM attachment. hPSCs have been previously cultured on laminin-coated^50,51^, vitronectin-coated^52,53^, and matrigel-coated^54^ stiff substrates and on very soft Matrigel-coated hydrogels of 100 Pa^55^. We here showed that only specific combinations of ECM proteins and substrate stiffness allow robust attachment and colony growth. This is consistent with studies on Matrigel on soft substrates^54,55^, since Matrigel is rich in laminins. We speculate that these protein-specific effects are mediated by the different force transduction characteristics of integrins that bind exclusively to vitronectin or laminin^56^. For mouse and for human embryonic stem cells alike, ECM–integrin interactions appear to be central to mechanoregulation of pluripotency.

For mouse naïve pluripotency, we found that low substrate stiffness was the most impactful factor increasing self-renewal. This is in line with some previous studies on stiffer substrates showing that intermediate levels of cell–fibronectin interactions^57^, low cell–ECM traction forces^9,10,58,59^ and limited cell spreading^25,60^ promote self-renewal. These factors are combined in our soft StemBond hydrogels, leading to an overall increase in self-renewal and higher expression of core transcription factors. Substrate stiffness has also been suggested to play a role in downstream differentiation of pluripotent stem cells to mesendoderm^26,54,61^, cardiomyocytes^62^, or neuronal^63^ cell types. here we found that mESCs underwent lineage commitment similarly on soft and stiff substrates. Thus, StemBond hydrogels are uniquely capable of supporting naïve pluripotency without restricting the differentiation potential of pluripotent stem cells.

The stabilisation of the mESC state was further corroborated with functional assays, showing increased self-renewal on soft substrates. A single pluripotency-driving signal (against two required on TCP or stiff substrates, among serum, LIF, CHIRON and PD03) was sufficient for maintenance of naïve pluripotency. Additionally, the doubling in reprogramming efficiency of mEpiSC in 2i suggest that soft substrates are not only permissive for the maintenance, but also for the acquisition, of naïve pluripotency. Bespoke microenvironments such as 3D matrices have previously been shown to accelerate cell reprogramming, in part through increased epigenetic remodelling^64^, but to our knowledge, this is the first time that 2D soft substrates are shown to improve reprogramming efficiency. In the sense that 2D substrates are more scalable and provide an easier platform for molecular biology assays, having 2D substrates that provide support at the level of 3D substrates could represent a significant advance in the field.

Based on our findings, we propose that soft substrates modulate activities of pathways regulating pluripotency. In particular, we found that ERK transcriptional activity on soft substrates is dampened, confirming ERK mechanosensitivity in mESCs as previously observed in cancer cells^21^, epidermal stem cells^12^ and mammary epithelial cells^65^. ERK targets showed similar stiffness-sensitivity across multiple conditions, both with and without LIF. Because ERK activity is decreased but not abolished, the observed effects on mESC self-renewal could be mediated either by threshold effects, or changes in the feedback loops regulating of ERK signalling^66^. Further investigations are needed to determine the upstream factors involved and the effect of soft substrates on ERK signalling dynamics.

Overall, we conclude that by promoting strong attachment but low cytoskeletal tension, soft StemBond hydrogels provide an optimal, well-defined microenvironment for long-term culture of mammalian pluripotent cells. They are as such well-suited for controlled studies of interplay between mechanical signalling and differentiation in stem cells. This work opens new possibilities to define alternative culture conditions with better control over stem cell fate decisions in physiologically mimetic conditions and with minimal need of small molecule inhibitors. Importantly, StemBond hydrogels can be adapted to match mechanical and adhesive properties other stem cell niches, as has been done recently to reverse the ageing of rat oligodendrocyte progenitor cells^15^. Future work will expand the scope of StemBond substrates to multiple stem cell types and culture systems.

## Methods

### Cell culture

E14 mESC and Rex1GFPd2 cells, a kind gift from Austin Smith’s laboratory at University of Cambridge, were cultured in either 10% Foetal Calf Serum + LIF medium (serum + LIF) or 2i+LIF medium following established protocols. Serum + LIF was supplemented with 2 mM l-glutamine (Gibco), MEM non-essential amino acids (Gibco), 1 mM Sodium Pyruvate (Invitrogen), and 0.1 mM 2-mercaptoethanol (Gibco). 2i + LIF was made up of N2B27 defined basal medium (1:1 Neurobasal and DMEM/F-12 medium (Invitrogen), 0.5% N2 (homemade), 1% B27 (ThermoFisher Scientific), 2 mM l-glutamine (Gibco), 0.1 mM 2-mercaptoethanol (Gibco) supplemented with MEK inhibitor (1 µM PD0325901), GSK3β inhibitor (3 µM CHIR99021) and/or 0.2 µg ml^−1^ murine LIF as indicated. Rex1+/*dGFP-IRES-bsd TetOn-Esrrb* + *CAG-rtTA3* (*iEsrrb*^40^) EpiSCs were cultured in N2B27 as above, supplemented with 12.5 ng ml^−1^ Fgf2, 20 ng ml^−1^ ActivinA (Hyvonen lab, Cambridge) and 6.25 µg ml^−1^ XAV 939 (Tocris). EpiSCs and reprogramming experiments were conducted in hypoxic conditions (7% CO_2_ and 5% O_2_).

Human embryo-derived naïve pluripotent cells (HNES1) and chemically reset naïve cells from a H9 background (cR-H9), both kindly gifted by Austin Smith’s laboratory, were maintained on irradiated MEFs in N2B27 medium supplemented with PDLGX (1 μM PD0325901, 10 ng ml^−1^ human LIF, 2 μM Gö6983, 2 μM XAV939), in 5% O_2_, 7% CO_2_ in a humidified chamber at 37°C, as described in^34^. Cells were passaged every 3–5 days using dissociation by Accutase. ROCK inhibitor (1 μg ml^−1^, Y-27632) and Geltrex (0.5 μl cm^−2^, Thermo Fisher Scientific) were added upon replating, with ROCK inhibitor removed the day after. Cells were maintained without MEFs in PDLGX media containing mouse purified laminin (10 μg cm^−2^, Merck) for one to two passages before seeding on hydrogels.

Human pluripotent stem cells (hPSC) Shef-6, a gift from Austin Smith’s laboratory, were cultured on TCP coated with truncated recombinant human vitronectin (Thermofisher, A14700) in E8 media (StemCell Technologies). Cells were dissociated in clumps with 0.5 mM EDTA every 4–7 days. For attachment studies, cells were seeded with ROCK inhibitor for one day to enable attachment to sulfo-SANPAH functionalised hydrogels.

MEFs were cultured in high-glucose DMEM containing 2 mM l-glutamine (Gibco), MEM non-essential amino acids (Gibco), 1 mM sodium pyruvate (Invitrogen), and 0.1 mM 2-mercaptoethanol (Gibco) on uncoated TCP dishes. Cells were passaged every 3–5 days for a maximum of 6 passages; cells were detached with PBS + EDTA and Accutase. To assess matrix stability, MEFs were seeded onto substrates coated with bovine rhodamine-fibronectin (#FNR01, Cytoskeleton). Mesoderm and neurectoderm differentiation were performed according to established protocols^32^. mESCs were plated in 2i on laminin-coated substrates. Media was changed the next day to N2B27. For mesoderm differentiation, after 1.5 days 20 ng ml^−1^ Activin A and 3 µM CHIR99021 were added to the media.

mESCs, iPSCs and EpiSCs and naïve hPSC cells were dissociated with accutase (Millipore) during passaging. Cells were plated on either TCP or hydrogel substrates coated with 200 µg ml^−1^ human plasma fibronectin (Corning, NY, USA and Millipore, Germany) at a density of 5000–15,000 cells/cm². hPSCs were plated on laminin-coated hydrogels (200 µg ml^−1^ Laminin, #CC095, Merck or 80 µg ml^−1^ Laminin 521, #A29249, Gibco) or Vitronectin-coated hydrogels (A14700, Thermofisher Scientific).

### Staining and imaging

Cell fixation, staining and slide mounting were done according to published protocols^67^ (see Table 3 for antibody list). Samples were imaged on Leica TCS SP5 confocal microscope, Zeiss LSM 710 confocal microscope, Zeiss 980 AiryScan microscope or Leica DMI 6000B Matrix HCS widefield microscope. Images were then analysed using Leica software and ImageJ. To assess gel surface coating, we used FITC-labelled BSA (Sigma-Aldrich).

In Fig. 1f, mean intensities were measured on each field of view and normalised to the maximal intensity observed in each batch. Background was measured for each substrate type on uncoated hydrogels, and the mean background values subtracted to the normalised intensities.

### Flow cytometry

The GFP signal in Rex1GFPd2 cells was monitored by flow cytometry on a Dako Cytomation CyAn ADP high-performance unit (Summit v4.3.02 software). Results were analysed using FlowJo and graphs were plotted using custom scripts in Matlab (Mathworks).

In Fig. 3d, cells were plated on high AHA StemBond hydrogels in serum + LIF and passaged every 48 h (keeping the same density at each passage).

### Hydrogel fabrication

Prior to hydrogel fabrication, coverslips were cleaned and functionalised with either Bind-Silane (GE Healthcare) for support coverslips or 20% Surfasil in chloroform (Fischer Scientific) for top coverslips. Hydrogel solutions were prepared according to Table 1 and polymerised between a support and a top coverslip for 15 min. The density of ligand binding sites was tuned by adapting the concentration of the co-polymer AHA (IUPAC name 2-(prop-2-enoylamino)hexanoic acid). Table 1 gives recipes of soft and stiff hydrogels for a final concentration of 48 mM AHA and A:B ratio of 25, which was used throughout the study, unless otherwise noted. After polymerisation, the top coverslips were removed and gels were rinsed twice in methanol, and soaked in PBS.

**Table 1:** Hydrogel recipes for 48 mM co-factor concentration.

*E* (kPa)	Acrylamide 40% (µl)	Bis-acrylamide 2% (µl)	AHA 2 M (µl)	H_2_O (µl)	TEMED (µl)	APS 10% (µl)
0.75	35	30	12	415.5	2.5	5
160	200	150	12	130.5	2.5	5

2 M AHA stock solution was prepared in methanol.

Hydrogels were then activated by 30 min treatment in 0.2 M EDAC (Sigma-Aldrich, Germany) + 0.5 M NHS (Sigma-Aldrich, Germany) in MES buffer, pH 6.1. They were coated with 200 µg ml^−1^ ECM protein diluted in HEPES buffer (50 mM pH 8.5) overnight at 4 °C. After washes, gels were blocked with 0.5 M Ethanolamine in HEPES buffer. Gels were stored at 4 °C until use.

Hydrogels without AHA were activated with sulfo-SANPAH (ThermoScientific, MA,USA). 1 mg ml^−1^ (unless otherwise noted) Sulfo-SANPAH was dissolved in HEPES, and activated by UV-light for up to 30 min.

### Alternative substrates

Elastic soft substrates of 1.5 kPa (# 81291, Ibidi, Germany) were coated with 30 µg ml^−1^ fibronectin following manufacturer’s guidelines.

### Atomic force microscopy

#### Substrate stiffness

Atomic Force Microscopy (AFM) was used to measure the elastic modulus of StemBond hydrogels, on a Nanowizard CellHesion 200 AFM (JPK Instruments) placed on an inverted microscope (Axio Observer A1, Zeiss) fitted with a motorised stage. Polystyrene beads (microParticles, Germany) with a diameter of 37.28 µm (for soft hydrogels) and 10.28 µm (for stiff hydrogels) were glued onto tipless silicon cantilevers (Arrow-TL1, Nanoworld, Switzerland) using MBond 610 Adhesive (Micro-Measurements). Spring constants of 0.03–0.07 N/m for soft hydrogels, 0.1–0.3 N/m for stiff hydrogels were determined by the thermal noise method. 10 force–distance curves at three different locations per gel (×5 replicate gels) were measured. Post-processing and analysis were done using JPK SPM data processing software, using the Hertz model to fit the approaching curve and extract the values of the substrate’s Young’s modulus.

#### Matrix tethering strength

Si–N gold-coated cantilevers with pyrex-nitride pyramidal tip (Nanoworld, #PNP-DB) were coated following an existing protocol^13,68^. Briefly probes were cleaned for 30 s in chloroform, then incubated with 5 M ethanolamine–HCl overnight. They were then washed in PBS and incubated for 30 min in 25 mM BS3 (bis(sulfosuccinimidyl)suberate, ThermoFisherScientific, #21580), washed again in PBS and immersed in 200 µg ml^−1^ antibody solution for 30 min. Finally, probes were rinsed and kept at 4 °C until use. Antibodies used were (rb) anti-Fibronectin (ABCAM, ab2413) and (rb) anti-IgG (Cell Signaling, #2729 S). Gels were prepared either with co-factor AHA or activated with sulfo-SANPAH. A control had no activation, and no coating. Activated gels were coated overnight with fibronectin at 200 µg ml^−1^ unless specified otherwise, then extensively rinsed. All gels were passivated with 1% BSA. Gels were stored in PBS at 4 °C until use. Two gels per condition were probed for both batches of measurements. Each sample was probed over three regions of 10 × 10 grids, points spaced by 10–20 µm. Head speed was 5 µm/s, loading rate ~160 nN/s setpoint was at 500 pN. Dwell time at the surface was 1–3 s before retraction. Data was processed using the JPK Data Processing Software (Version 6.1.86 and 6.1.131) and Matlab (Mathworks). Briefly, for each measurement, we plotted the rupture force (minimal value of the vertical deflection) against the rupture length (see Supplementary Fig. 1b). To rule out non-specific interactions, we used probes coated with anti-IgG. Data on stiff and soft substrates were analysed separately because of differences in sample/probe interaction area (setpoint was kept constant at 500 pN). Processing then followed these steps:

(i) For each curve, rupture force and rupture length are extracted.
(ii) A threshold of both rupture force and rupture length were set at the median of the distributions for negative controls.
(iii) The 2-d density map of negative controls and of samples were computed (Supplementary Fig. 1c). The regions where the density of samples was more than twice that of controls were defined as clusters of significant events, after a median filtering to smooth the cluster (Supplementary Fig. 1d).
(iv) For all the significant binding events, the mean rupture force is determined (see Fig. 1e). Graphs show average over 2–4 samples, errors are standard error of the mean. Total number of measurements per sample ranged from 34 to 696 and number of significant events per sample ranged from 0 to 233.

### Gene expression assays

For gene expression analysis, cells were lysed in RLT buffer (QIAGEN) and RNA extracted using RNEasy extraction kit (Qiagen). RT-qPCR was performed using Superscript Transcriptase III (ThermoScientific, MA, USA) and gene expression was then assessed using the relevant Taqman probes (FAM) (Table 2) and Gapdh (VIC) as endogenous control.

**Table 2:** List of probes for gene expression assays.

Gene	Cat. no.	Company
Esrrb	Mm00442411_m1	ThermoFisher Scientific
Gapdh (Endogenous Control)	4352339E	ThermoFisher Scientific
Nanog	Mm02384862_g1 and Hs02387400_m1	ThermoFisher Scientific
Lefty2	Mm00774547_m1	ThermoFisher Scientific
Klf4	Mm00516104_m1 and Hs00358836_m1	ThermoFisher Scientific
Klf2	Mm00500486_g1	ThermoFisher Scientific
Nr0b1	Mm00431729_m1	ThermoFisher Scientific
Pou5f1	Mm00658129_gH and Hs04260367_qH	ThermoFisher Scientific
Tfcp2l1	Mm00470119_m1 and Hs00232708_m1	ThermoFisher Scientific
Zfp42	Mm03053975_g1	ThermoFisher Scientific
Sox1	Mm00486299_s1	ThermoFisher Scientific
Pax6	Mm00443081_m1	ThermoFisher Scientific
Foxa2	Mm01976556_s1	ThermoFisher Scientific
Eomes	Mm01351984_m1	ThermoFisher Scientific
T	Mm00436877_m1	ThermoFisher Scientific
Fgf5	Mm00438919_m1	ThermoFisher Scientific
Klf17	Hs00702999_m1	ThermoFisher Scientific

### RNA sequencing and analysis

Library preparation was done by in-house facility using Pico mammalian V2 (Takara, USA), NuGen (NuGen, CA, USA) or RiboZero and Nextflex (Bioo Scientific, TX, USA) kits. Sequencing was performed on Illumina HiSeq4000 yielding 350 Million reads per lane.

### RNA sequencing data processing, transcriptome analysis and network analysis

#### Dataset of Fig. 6a, b

Mouse genome build GRCm38/mm10 were used to align reads with GSNAP version 2015-09-29^69^. Genes were annotated using Ensembl release 81^70^ and read counts were quantified using HTSeq^71^. Differential expression analysis was computed using DESeq2^72^ on protein-coding genes, from pairwise comparisons of samples with either same AHA concentration or same stiffness.

DAVID 6.7^73,74^ was used to compute the statistical enrichment of Gene Ontology terms, using genes up- and down-regulated by stiffness as input. Cytoscape^75^ and the enrichment map plugin^76^ were used for network construction and visualisation (Supplementary Fig. 7b). Node size is scaled by the number of genes contributing to over-representation of biological processes; edges are plotted in width proportional to the overlap between gene sets. Node colour represents the different AHA conditions in which each biological process is significantly enriched.

#### Dataset of Fig. 6c–e

Mouse genome build GRCm38/mm10 were used to align reads with STAR 2.5.2a^77^. Genes were annotated using mouse annotation from Ensembl release 87^78^ and splice junction donor/acceptor overlap settings were tailored to the read length of each dataset. Alignments to gene loci were quantified with Htseq-count^71^ based on annotation from Ensembl 87.

Principal component analyses was performed based on log_2_ FPKM values computed with the Bioconductor packages DESeq^79^, FactoMineR^80^ in addition to custom scripts. In addition, DESeq was used to perform differential analysis between stiff and soft substrates.

In order to identify genes with the greatest expression variability we fitted a non-linear regression curve between average log_2_ FPKM and the square of coefficient of variation. Specific thresholds were applied along the *x*-axis (average log_2_ FPKM) and *y*-axis (log CV^2^) to identify the most variable genes.

DAVID 6.8^73,74^ was used to compute the statistical enrichment of Gene Ontology terms, using modulated genes in Serum-only conditions as input (Fig. 6d and Supplementary Fig. 8d).

STRING database (https://string-db.org/)^81^ was used to retrieve gene–gene interaction and Cytoscape was used to visualise the resulting network (Supplementary Fig. 8b). Transcription factor and transcription co-factor annotation were downloaded from AnimalTFDB (http://bioinfo.life.hust.edu.cn/AnimalTFDB/).

Cytoscape^75^ and the enrichment map plugin^76^ were used for network construction and visualisation (Supplementary Fig. 8c). Node size is scaled by the number of genes contributing to over-representation of biological processes; edges are plotted in width proportional to the overlap between gene sets. The colour represents the centred percentage of up/downregulated genes for each biological process.

#### Dataset of Fig. 6f and Supplementary Figs. 9, 10

Mouse genome build GRCm38/mm10 were used to align reads with Tophat v2.1.0^82^. Genes were annotated using Ensembl release 86^78^ and read counts were quantified using Featurecount v1.5.0^83^. Differential expression analysis was computed using DESeq2^72^ on protein coding genes.

### Clonogenicity assays and alkaline phosphatase staining

For clonogenicity assays of mESCs in serum + LIF, 50,000 cells were plated on TCP or hydrogels coated with fibronectin in the indicated medium for 5 days (Fig. 4a(i) and b). For clonogenicity assays following multiple passaging in N2B27 + CHIRON and N2B27 + PD03, cells were cultured on the hydrogels for three passages by serial dilution or keeping constant plating density as indicated in Fig. 4a and in figure legends. Then 400 cells/well (Fig. 4c) or 1000 cells/well (Fig. 4d) were replated on gelatin-coated TCP in 2i + LIF for a minimum of 5 days. For clonogenicity assays of naïve hPSCs, cells were passaged at a 1:5 dilution onto TCP or hydrogels coated with mouse laminin (#CC095, Merck) in the indicated medium for 5–6 days. Cells were then dissociated to single cells and replated at 1250 cells/well on irradiated MEFs in TCP plates, with PDLGX media supplemented with ROCK inhibitor and Geltrex, for 6 days.

Cells were subsequently fixed in 8% formaldehyde and stained using the Alkaline-Phosphatase kit (86R 1KT, Sigma-Aldrich, Germany) following the manufacturer’s protocol. Images were acquired using CellSens software and an X-51 Olympus microscope system with motorised stage and camera, ×4 magnification. Colonies were then segmented and counted using ImageJ with manual verification at each step. In some wells there were colonies lining the side of the coverslip but beneath the hydrogel and these were therefore not counted.

### Chimaeras

This research has been regulated under the Animals (Scientific Procedures) Act 1986 Amendment Regulations 2012 following ethical review by the University of Cambridge Animal Welfare and Ethical Review Body (AWERB). Use of animals in this project was approved by the ethical review committee for the University of Cambridge, and relevant Home Office licences (Project license No. 80/2597) are in place.

Rosa26-CreERT2^+/+ ^mESC from a C57BL/6-Agouti background (gift from Koo lab) were first transfected with a piggyback transposon vector (PB-GFP). Cells were then cultured in N2B27 + PD03 on soft hydrogels for three passages by serial dilution of 1:2 at each passage. The cells were then dissociated into single cells, before injection into C57BL/6 host blastocysts at stage E3.5. Contribution of the injected cells to the mice is reflected by GFP expression. 5 out of 6 pups from the first generation of chimaeras had significant GFP expression. 7 pups from the second generation expressed GFP (Fig. 4e), demonstrating germline contribution. The full quantification is given in Supplementary Table 1. Strains used: C57BL/6 for host embryos and stud males, CBAB6F1 for recipient females, CD1 for the vasectomised males. All animals were housed between 18 and 22 °C, 40% and 60% humidity and a 12 h day/night light cycle, with food and water supplied ad libitum.

### Reprogramming of mEpiSCs

10,000 EpiSCs per well (six-well plate) were plated in N2B27 + Fgf2+ActivinA+XAV medium, on the fibronectin-coated TCP and hydrogels. 24 h later, reprogramming was induced by medium switch to N2B27 + 2i + 1 µg ml^−1^ doxycycline (MP Biomedicals). After 4 days, dox-induction of *iEsrrb* was withdrawn and 20 μg ml^−1^ blasticidin (Gibco) was applied to select for *Rex1*::dGFP-IRES-bsd reporter activity. On day 8, ×4 images were acquired using CellSens software and an X-51 Olympus microscope system with motorised stage and camera. iPSC colonies with active *Rex1* reporter were counted manually. Resultant iPSCs were passaged once onto TCP then RNA lysates were harvested.

### Western blots

Cells were lysed in RIPA buffer (Cell Signaling, Leiden, The Netherlands) and spun down, then sonicated for 1.5 min. The supernatant was then denatured in SDS buffer at 95 °C for 5 min. Mini-protean gels (12% or gradient 8–14%, Bio-Rad) were used for gel running. Protein gels were then transferred onto a nitrocellulose membrane using a standard wet transfer procedure. Membranes were subsequently blocked with 5% BSA in TBS-Tween 20 for 2 h, before overnight staining with primary antibodies at 4 °C. Secondary antibodies incubated for 1 h at room temperature. ECL Fire (ThermoScientific, MA, USA) and ECL Prime (GE Healthcare) reagents were used to reveal the blots. See Table 3 for antibody list.

**Table 3:** List of antibodies.

Antibody	Cat. no.	Company	Lot number	Dilution
Phospho ERK	4370S	Cell Signaling	#17 Ref:05/2016 #17 Ref:01/2017	1:1000 (WB), 1:200 (IF)
Phospho ERK	9106	Cell Signaling		1:1000 (WB)
ERK	4695S	Cell Signaling	#21 Ref:05/2016	1:1000 (WB)
Phospho Stat3 (m)	4113S	Cell Signaling	#5 Ref:07/2016	1:1000 (WB)
Phospho Stat3 (rb)	9145	Cell Signaling	#22 Ref:12/2013	1:1000 (WB)
Stat3	9139S	Cell Signaling	#10 Ref:12/2016	1:1000 (WB)
Esrrb (m)	PP-H6705	Perseus Proteomics	A2	1:500 (WB)
Nanog (rb)	A300-398A	Bethyl Laboratories Inc.		1:2000 (WB)
H3 (rb)	ab1791	Abcam		1:5000 (WB)
Gapdh (m)	97166S	Cell Signaling	#3 Ref:05/2017	1:2000 (WB)
Gapdh (rb)	5174S	Cell Signaling	#6 Ref:11/2016	1:2000 (WB)
Anti-rb HRP	7074S	Cell Signaling	#26 Ref:12/2016	1:2500 (WB)
Anti-m HRP	7076S	Cell Signaling	#32 Ref:12/2015	1:2500 (WB)
Phospho Paxillin	2541	Cell Signaling	#6 Ref:08/2015	1 :50 (IF)
Integrin ß1 (CD29) (rat)	553715	BD Biosciences		1:100 (IF)
Phalloidin 555	8953	Cell Signaling	#3 Ref:11/2016	1 :20 (IF)
Sox1 (rb)	4194S	Cell Signaling	#2 Ref 03/2019	1:100 (IF)
T/Brachyury	AF2085	R&D Systems	Lot KQP0315041	1:400 (IF)
Fibronectin (rb)	ab199056	Abcam		1:1000 (IF)
Fibronectin (rb)	ab2413	Abcam		200 µg ml^−1^ (AFM) 1:500 (IF)
Anti-IgG (rb)	2729S	Cell Signaling		200 µg ml^−1^ (AFM)
Goat anti-rabbit 680	A21109	Thermofisher		1:20,000 (WB)
Goat anti-mouse 790	A11357	Thermofisher		1:20,000 (WB)

### Statistics and reproducibility

In all figures, error bars show standard deviation (std) over *n* independent replicates (indicated in legend), unless otherwise indicated.

Statistical differences across substrate conditions were determined using the ANOVA test on independent samples (tests done using Matlab). In most figures, tests are designed to compare across different types of substrate (TCP, Stiff and Soft hydrogels); in Figs. 1e and 2a, multiple adhesiveness levels are included in the design. Only *p*-value corresponding to individual genes are reported (we do not compare expression between different genes). Results are indicated in the figures with absolute *p*-values or *p* < 0.001 for very small *p*-values. n.s. means no statistically significant differences were found. For Supplementary Fig. 9b–d, a permutation test was used to determine if the expression of a selection of genes was systematically different on stiff versus soft substrates.

For experiments where representative images are shown, experiments were repeated twice (Figs. 4f, 5b, and Supplementary Figs. 1a, 2c, 3a, b, 4c) or three times (Fig. 2b, d, and Supplementary Figs. 5a, e, 9a) with two biological repeats or two gel samples each time.

All results of statistical tests are given in the Source Data File.

### Estimation of surface density of adhesive ligand anchoring points

The surface density σ of anchoring points was estimated by computing the number of molecules in a unit cubic volume from the concentration (volumetric density) and then extracting the corresponding number of molecules on the surface of this cubic volume. σ is given by the following formula:

$\sigma ={\left({N}_{{\mathrm {A}}}\cdot \left[{{ {AHA}}}\right]\right)}^{2/3}\cdot {10}^{-10}$ (Nsites µm^−^²) where *N*_A_ is the Avogadro constant, and [AHA] is the molar concentration of AHA.

### Reporting summary

Further information on research design is available in the Nature Research Reporting Summary linked to this article.

## Supplementary information

Supplementary Information

Description of Additional Supplementary Files

Supplementary Dataset 1

Supplementary Dataset 2

Supplementary Dataset 3

Supplementary Dataset 4

Reporting Summary

## Data Availability

All RNA-sequencing data generated in this study have been deposited in the GEO database under accession code GSE125617.  Source data are provided with this paper.

## Source data

Source Data

## References

[1] Crowder SW, Leonardo V, Whittaker T, Papathanasiou P, Stevens MM (2016). Material cues as potent regulators of epigenetics and stem cell function. Cell Stem Cell.

[2] Lutolf MP, Gilbert PM, Blau HM (2009). Designing materials to direct stem-cell fate. Nature.

[3] Sun Y, Chen CS, Fu J (2012). Forcing stem cells to behave: a biophysical perspective of the cellular microenvironment. Annu. Rev. Biophys..

[4] Damljanović V, Lagerholm BC, Jacobson K (2005). Bulk and micropatterned conjugation of extracellular matrix proteins to characterized polyacrylamide substrates for cell mechanotransduction assays. Biotechniques.

[5] Grevesse T, Versaevel M, Circelli G, Desprez S, Gabriele S (2013). A simple route to functionalize polyacrylamide hydrogels for the independent tuning of mechanotransduction cues. Lab Chip.

[6] Kandow CE, Georges PC, Janmey PA, Beningo KA (2007). Polyacrylamide hydrogels for cell mechanics: steps toward optimization and alternative uses. Methods Cell Biol..

[7] Engler AJ, Sen S, Sweeney HL, Discher DE (2006). Matrix elasticity directs stem cell lineage specification. Cell.

[8] Park JS (2011). The effect of matrix stiffness on the differentiation of mesenchymal stem cells in response to TGF-β. Biomaterials.

[9] Chowdhury F (2010). Soft substrates promote homogeneous self-renewal of embryonic stem cells via downregulating cell-matrix tractions. PLoS ONE.

[10] Lü D, Luo C, Zhang C, Li Z, Long M (2014). Differential regulation of morphology and stemness of mouse embryonic stem cells by substrate stiffness and topography. Biomaterials.

[11] Xia S, Yim EKF, Kanchanawong P (2019). Molecular organization of integrin-based adhesion complexes in mouse embryonic stem cells. ACS Biomater. Sci. Eng..

[12] Trappmann B (2012). Extracellular-matrix tethering regulates stem-cell fate. Nat. Mater..

[13] Wen JH (2014). Interplay of matrix stiffness and protein tethering in stem cell differentiation. Nat. Mater..

[14] Barriga EH, Franze K, Charras G, Mayor R (2018). Tissue stiffening coordinates morphogenesis by triggering collective cell migration in vivo. Nature.

[15] Segel M (2019). Niche stiffness underlies the ageing of central nervous system progenitor cells. Nature.

[16] Ying Q-L (2008). The ground state of embryonic stem cell self-renewal. Nature.

[17] Wray J, Kalkan T, Smith AG (2010). The ground state of pluripotency. Biochem. Soc. Trans..

[18] Niwa H, Burdon T, Chambers I, Smith AG (1998). Self-renewal of pluripotent embryonic stem cells is mediated via activation of STAT3. Genes Dev..

[19] Matsuda T (1999). STAT3 activation is sufficient to maintain an undifferentiated state of mouse embryonic stem cells. EMBO J..

[20] Toyooka Y, Shimosato D, Murakami K, Takahashi K, Niwa H (2008). Identification and characterization of subpopulations in undifferentiated ES cell culture. Development.

[21] Paszek MJ (2005). Tensional homeostasis and the malignant phenotype. Cancer Cell.

[22] Lammerding J, Kamm RD, Lee RT (2004). Mechanotransduction in cardiac myocytes. Ann. N. Y. Acad. Sci..

[23] Li D (2010). Integrated biochemical and mechanical signals regulate multifaceted human embryonic stem cell functions. J. Cell Biol..

[24] Fernández-Sánchez ME (2015). Mechanical induction of the tumorigenic β-catenin pathway by tumour growth pressure. Nature.

[25] Murray P (2013). The self-renewal of mouse embryonic stem cells is regulated by cell-substratum adhesion and cell spreading. Int. J. Biochem. Cell Biol..

[26] Evans ND (2009). Substrate stiffness affects early differentiation events in embryonic stem cells. Eur. Cells Mater..

[27] Chowdhury F (2010). Material properties of the cell dictate stress-induced spreading and differentiation in embryonic stem cells. Nat. Mater..

[28] Pless DD, Lee YC, Roseman S, Schnaar RL (1983). Specific cell adhesion to immobilized glycoproteins demonstrated using new reagents for protein and glycoprotein immobilization. J. Biol. Chem..

[29] Yip AK (2013). Cellular response to substrate rigidity is governed by either stress or strain. Biophys. J..

[30] Wang X (2018). Characterizing inner pressure and stiffness of trophoblast and inner cell mass of blastocysts. Biophys. J..

[31] Kalkan T (2017). Tracking the embryonic stem cell transition from ground state pluripotency. Development.

[32] Mulas C, Kalkan T, Smith A (2017). NODAL secures pluripotency upon embryonic stem cell progression from the ground state. Stem Cell Rep..

[33] Guo G (2017). Epigenetic resetting of human pluripotency. Development.

[34] Rostovskaya, M., Stirparo, G. G. & Smith, A. Capacitation of human naïve pluripotent stem cells for multi-lineage differentiation. *Development*. **146**, dev172916 (2019). 10.1242/dev.172916 PMC6467473 30944104

[35] Brons IGM (2007). Derivation of pluripotent epiblast stem cells from mammalian embryos. Nature.

[36] Tesar PJ (2007). New cell lines from mouse epiblast share defining features with human embryonic stem cells. Nature.

[37] Guo G (2009). Klf4 reverts developmentally programmed restriction of ground state pluripotency. Development.

[38] Festuccia N (2012). Esrrb is a direct Nanog target gene that can substitute for Nanog function in pluripotent cells. Cell Stem Cell.

[39] Stuart HT (2014). NANOG amplifies STAT3 activation and they synergistically induce the naive pluripotent program. Curr. Biol..

[40] Stuart HT (2019). Distinct molecular trajectories converge to induce naive pluripotency. Cell Stem Cell.

[41] Kinoshita M, Smith A (2018). Pluripotency deconstructed. Dev. Growth Differ..

[42] Kunath T (2007). FGF stimulation of the Erk1/2 signalling cascade triggers transition of pluripotent embryonic stem cells from self-renewal to lineage commitment. Development.

[43] Nichols J, Smith AG (2009). Naive and primed pluripotent states. Cell Stem Cell.

[44] Shahbazi MN (2017). Pluripotent state transitions coordinate morphogenesis in mouse and human embryos. Nature.

[45] Boroviak T (2015). Lineage-specific profiling delineates the emergence and progression of naive pluripotency in mammalian embryogenesis. Dev. Cell.

[46] Lee JP, Kassianidou E, MacDonald JI, Francis MB, Kumar S (2016). N-terminal specific conjugation of extracellular matrix proteins to 2-pyridinecarboxaldehyde functionalized polyacrylamide hydrogels. Biomaterials.

[47] Lakins JN, Chin AR, Weaver VM (2012). Exploring the link between human embryonic stem cell organization and fate using tension-calibrated extracellular matrix functionalized polyacrylamide gels. Methods Mol. Biol..

[48] Loebel C, Mauck RL, Burdick JA (2019). Local nascent protein deposition and remodelling guide mesenchymal stromal cell mechanosensing and fate in three-dimensional hydrogels. Nat. Mater..

[49] Khetan S (2013). Degradation-mediated cellular traction directs stem cell fate in covalently crosslinked three-dimensional hydrogels. Nat. Mater..

[50] Xu C (2001). Feeder-free growth of undifferentiated human embryonic stem cells. Nat. Biotechnol..

[51] Rodin, S. et al. Clonal culturing of human embryonic stem cells on laminin-521/E-cadherin matrix in defined and xeno-free environment. *Nat. Commun.* **5**, 3195 (2014). 10.1038/ncomms4195 24463987

[52] Braam SR (2008). Recombinant vitronectin is a functionally defined substrate that supports human embryonic stem cell self-renewal via αVβ5 integrin. Stem Cells.

[53] Chen G (2011). Chemically defined conditions for human iPSC derivation and culture. Nat. Methods.

[54] Przybyla L, Lakins JN, Weaver VM (2016). Tissue mechanics orchestrate Wnt-dependent human embryonic stem cell differentiation. Cell Stem Cell.

[55] Keung AJ, Asuri P, Kumar S, Schaffer DV (2012). Soft microenvironments promote the early neurogenic differentiation but not self-renewal of human pluripotent stem cells. Integr. Biol..

[56] Seetharaman S, Etienne-Manneville S (2018). Integrin diversity brings specificity in mechanotransduction. Biol. Cell.

[57] Hunt GC, Singh P, Schwarzbauer. JE (2012). Endogenous production of fibronectin is required for self-renewal of cultured mouse embryonic stem cells. Exp. Cell Res..

[58] Hayashi Y (2007). Integrins regulate mouse embryonic stem cell self-renewal. Stem Cells.

[59] Uda Y (2011). Force via integrins but not E-cadherin decreases Oct3/4 expression in embryonic stem cells. Biochem. Biophys. Res. Commun..

[60] De Belly H (2021). Membrane tension gates ERK-mediated regulation of pluripotent cell fate. Cell Stem Cell.

[61] Candiello, J., Singh, S. S., Task, K., Kumta, P. N. & Banerjee, I. Early differentiation patterning of mouse embryonic stem cells in response to variations in alginate substrate stiffness. *J. Biol. Eng.* **7**, 9 (2013). 10.1186/1754-1611-7-9 PMC3643844 23570553

[62] Arshi A (2013). Rigid microenvironments promote cardiac differentiation of mouse and human embryonic stem cells. Sci. Technol. Adv. Mater..

[63] Ali S, Wall IB, Mason C, Pelling AE, Veraitch FS (2015). The effect of Young’s modulus on the neuronal differentiation of mouse embryonic stem cells. Acta Biomater..

[64] Caiazzo M (2016). Defined three-dimensional microenvironments boost induction of pluripotency. Nat. Mater..

[65] Provenzano PP, Inman DR, Eliceiri KW, Keely PJ (2009). Matrix density-induced mechanoregulation of breast cell phenotype, signaling and gene expression through a FAK–ERK linkage. Oncogene.

[66] Nett IR, Mulas C, Gatto L, Lilley KS, Smith A (2018). Negative feedback via RSK modulates Erk‐dependent progression from naïve pluripotency. EMBO Rep..

[67] Agley CC, Rowlerson AM, Velloso CP, Lazarus NR, Harridge SDR (2013). Human skeletal muscle fibroblasts, but not myogenic cells, readily undergo adipogenic differentiation. J. Cell Sci..

[68] Chirasatitsin, S. & Engler, A. J. Detecting cell-adhesive sites in extracellular matrix using force spectroscopy mapping. *J. Phys. Condens. Matter*. **22**, 194102 (2010). 10.1088/0953-8984/22/19/194102 PMC2997741 21152375

[69] Wu TD, Nacu S (2010). Fast and SNP-tolerant detection of complex variants and splicing in short reads. Bioinformatics.

[70] Cunningham F (2015). Ensembl 2015. Nucleic Acids Res..

[71] Anders S, Pyl PT, Huber W (2015). HTSeq-a Python framework to work with high-throughput sequencing data. Bioinformatics.

[72] Love MI, Huber W, Anders S (2014). Moderated estimation of fold change and dispersion for RNA-seq data with DESeq2. Genome Biol..

[73] Huang DW, Sherman BT, Lempicki RA (2009). Systematic and integrative analysis of large gene lists using DAVID bioinformatics resources. Nat. Protoc..

[74] Huang DW, Sherman BT, Lempicki RA (2009). Bioinformatics enrichment tools: paths toward the comprehensive functional analysis of large gene lists. Nucleic Acids Res..

[75] Shannon P (2003). Cytoscape: a software environment for integrated models of biomolecular interaction networks. Genome Res..

[76] Isserlin R, Merico D, Voisin V, Bader GD (2014). Enrichment Map—a Cytoscape app to visualize and explore OMICs pathway enrichment results. F1000Research.

[77] Dobin A (2013). STAR: ultrafast universal RNA-seq aligner. Bioinformatics.

[78] Yates A (2016). Ensembl 2016. Nucleic Acids Res..

[79] Anders S, Huber W (2010). Differential expression analysis for sequence count data. Genome Biol..

[80] Lê S, Josse J, Husson F (2008). FactoMineR: an R package for multivariate analysis. J. Stat. Softw..

[81] Snel B, Lehmann G, Bork P, Huynen MA (2000). STRING: a web-server to retrieve and display the repeatedly occurring neighbourhood of a gene. Nucleic Acids Res..

[82] Kim D (2013). TopHat2: accurate alignment of transcriptomes in the presence of insertions, deletions and gene fusions. Genome Biol..

[83] Liao Y, Smyth GK, Shi W (2014). featureCounts: an efficient general purpose program for assigning sequence reads to genomic features. Bioinformatics.

