# Supplementary material for: StemBond hydrogels control the mechanical microenvironment for pluripotent stem cells
Source: Nat Commun. 2021 Oct 21;12:6132. doi: 10.1038/s41467-021-26236-5 (PMC8531294; doi:10.1038/s41467-021-26236-5)
Supplement: Supplementary file 1 — Supplementary Information [file 41467_2021_26236_MOESM1_ESM.pdf]

SUPPLEMENTARY INFORMATION

StemBond hydrogels control the mechanical microenvironment for pluripotent stem cells

Céline Labouesse[1 †], Bao Xiu Tan[1,2 †], Chibeza C. Agley[1 †], Moritz Hofer[1], Alexander K. Winkel[3], Giuliano G. Stirparo[1], Hannah T. Stuart[1,4], Christophe M. Verstreken[1,2], Carla Mulas[1], William Mansfield[1], Paul Bertone[1], Kristian Franze[3,4,5], Jose C. R. Silva[1,6*] and Kevin J. Chalut[1,2*‡]

Supplementary Table 1: Quantification of chimaera experiments (related to Figure 4e)

Generation	Viable pups at birth (m/f)	GFP positive (m/f)	GFP negative (m/f)	Untested (m/f)
F0	11 (8/3)	8 (7/1)	3 (1/2)	
F1	64 (19/45)	24 (5/19)	20 (3/17)	20 (11/9)

Supplementary Figure 1

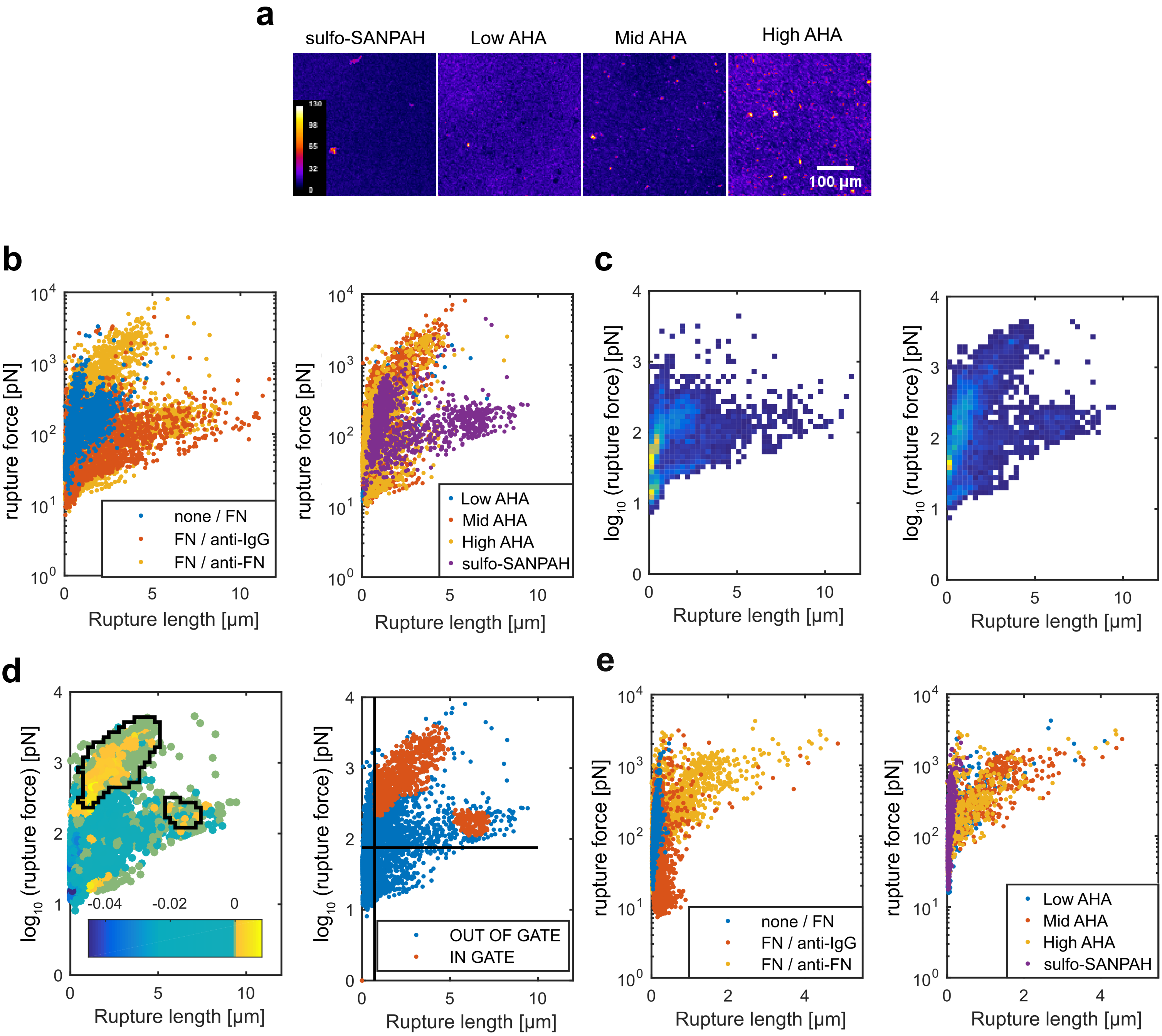

Supplementary Figure 1 (in relation to Figure 1): StemBond hydrogels increase the stability of the ECM layer

a – Fibronectin coverage on stiff StemBond hydrogels in absence of cells. There are no significant differences in fluorescence intensity between conditions.

b – Plots showing rupture force vs rupture length for all measured curves on soft hydrogels. Each point corresponds to one measurement. (Left) Comparison of measurements for substrates coated with either fibronectin ('FN') or no matrix protein ('none') and probed with a cantilever functionalised either with anti-Fibronectin ('anti-FN') or anti-IgG antibody. Anti-IgG antibody serves to detect non-specific binding events. (Right) Comparison of measurements for different substrate functionalisation between 1mg.ml^{-1} sulfo-SANPAH or Low, Mid and High AHA StemBond hydrogels.

c – Density maps of the all measurements for negative controls (left) and real samples (right).

d – Same data as in b. (Left) The colour code shows the amplitude of density (samples)-2xdensity(controls). This was used to identify clusters of points where sample measurements did not overlap with negative controls. The clusters of significant events are outlined in black (see Methods for details). (Right) Similar to the left panel, red points are within the clusters of significant events, blue points are out and discarded for following steps. Vertical and Horizontal lines show the median of rupture length and rupture force of negative controls, used as thresholds.

e – Plots showing rupture force vs rupture length for all measured curves on stiff hydrogels. Each point corresponds to one measurement. (Left) Comparison of measurements for substrates coated with either fibronectin ('FN') or no matrix protein ('none') and probed with a cantilever functionalised either with anti-Fibronectin ('anti-FN') or anti-IgG antibody. Anti-IgG antibody serves to detect non-specific binding events. (Right) Comparison of measurements for different substrate functionalisation between 1mg.ml^{-1} sulfo-SANPAH or Low, Mid and High AHA StemBond hydrogels.

Supplementary Figure 2

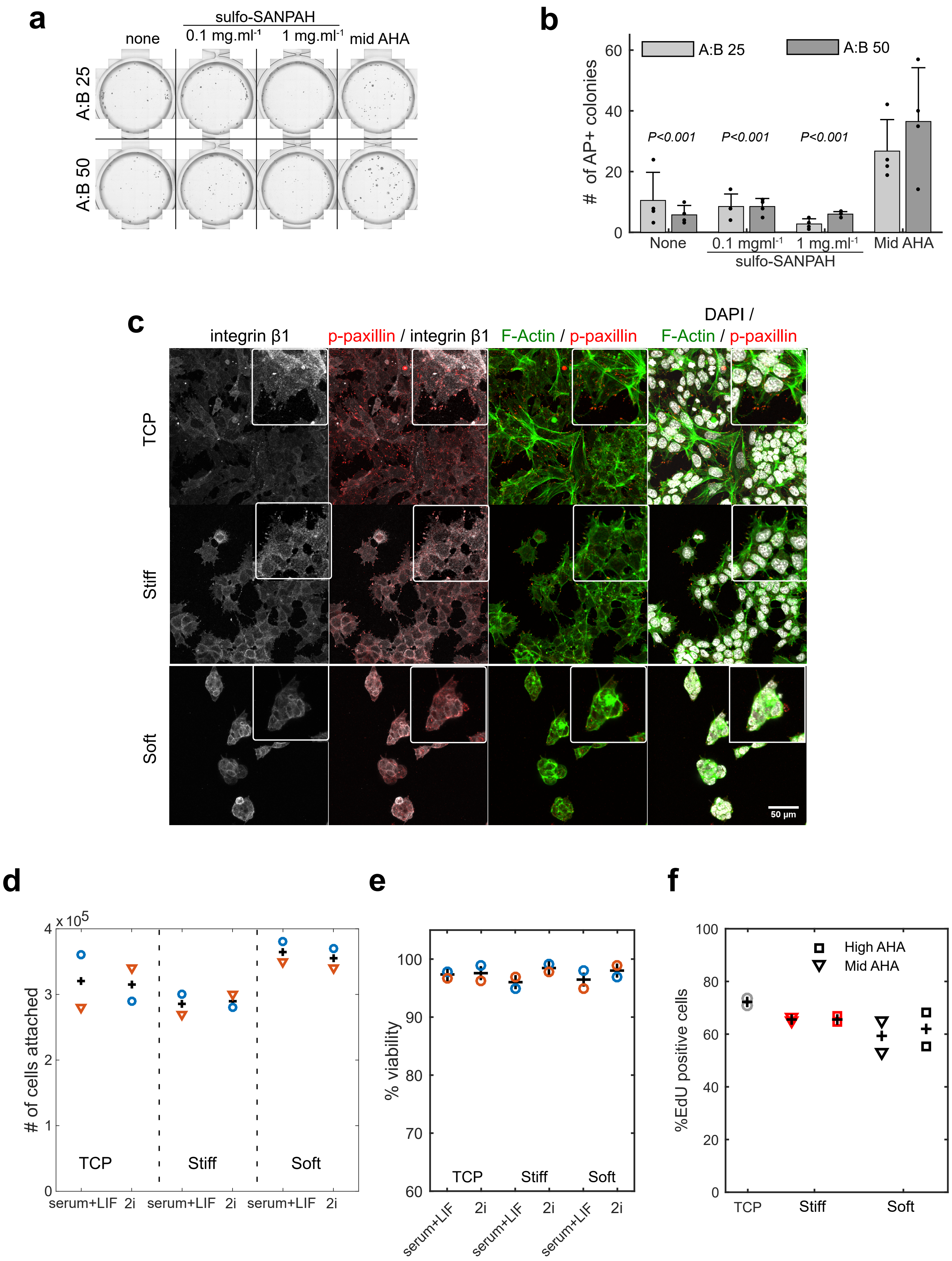

Supplementary Figure 2 (in relation to Figure 2): mESCs show strong attachment, spreading and viability on StemBond hydrogels

a – Snapshots of Alkaline-Phosphatase (AP) staining of ES colonies on soft hydrogels. Hydrogels were prepared with an acrylamide:bis-acrylamide ratio of either 25 (top row) or 50 (bottom row), with different surface functionalisations. All hydrogels (including the unfunctionalised ones) were coated with 200µg.ml^{-1} of fibronectin. Cells were seeded at clonal density for 6 days before fixation and staining.

b – Mean +/- standard deviation of counts of number of Alkaline-Phosphatase (AP) positive colonies for different surface functionalisation methods (n = 4 independent samples). P-values were computed using a one-way ANOVA with Tukey-Kramer's multiple comparison test, comparing the number of naïve colonies robustly attached to the substrate for different functionalisation methods to Mid AHA StemBond hydrogels. Acrylamide:bis-acrylamide ratio (A:B) was not a significant factor.

c – Immunostaining for integrin β1 (gray), phospho-paxillin (red), F-actin (green) and DAPI (gray) cells on tissue culture plastic (TCP), soft or stiff mid-AHA hydrogels. Focal adhesions marked by integrin β1 and phospho-paxillin foci are only visible on TCP and stiff substrates. Scale bar: 50µm. Zoom in insets are 80µm x 80µm.

d – Cell attachment on fibronectin-coated tissue culture plastic (TCP), stiff hydrogels and soft mid AHA hydrogels. 175'000 cells were plated for 24hrs in culture in 2i or serum+LIF medium before being detached and counted (n = 2 independent samples). 'o' and 'Δ' show individual data points, '+' show the mean.

e – Percentage of viable cells assessed by Trypan Blue staining counted on flow cytometer (ViCell) after 24 hrs on different substrates. Individual data points 'o' and mean '+' (n = 2 independent samples).

f – Percentage of EdU positive cells. Cells were seeded overnight in 2i on gels, then incubated for 25min with EdU before fixing and quantification by imaging. 'Δ' and '○' show individual measurements, '+' show the mean (n = 2 independent samples).

Supplementary Figure 3

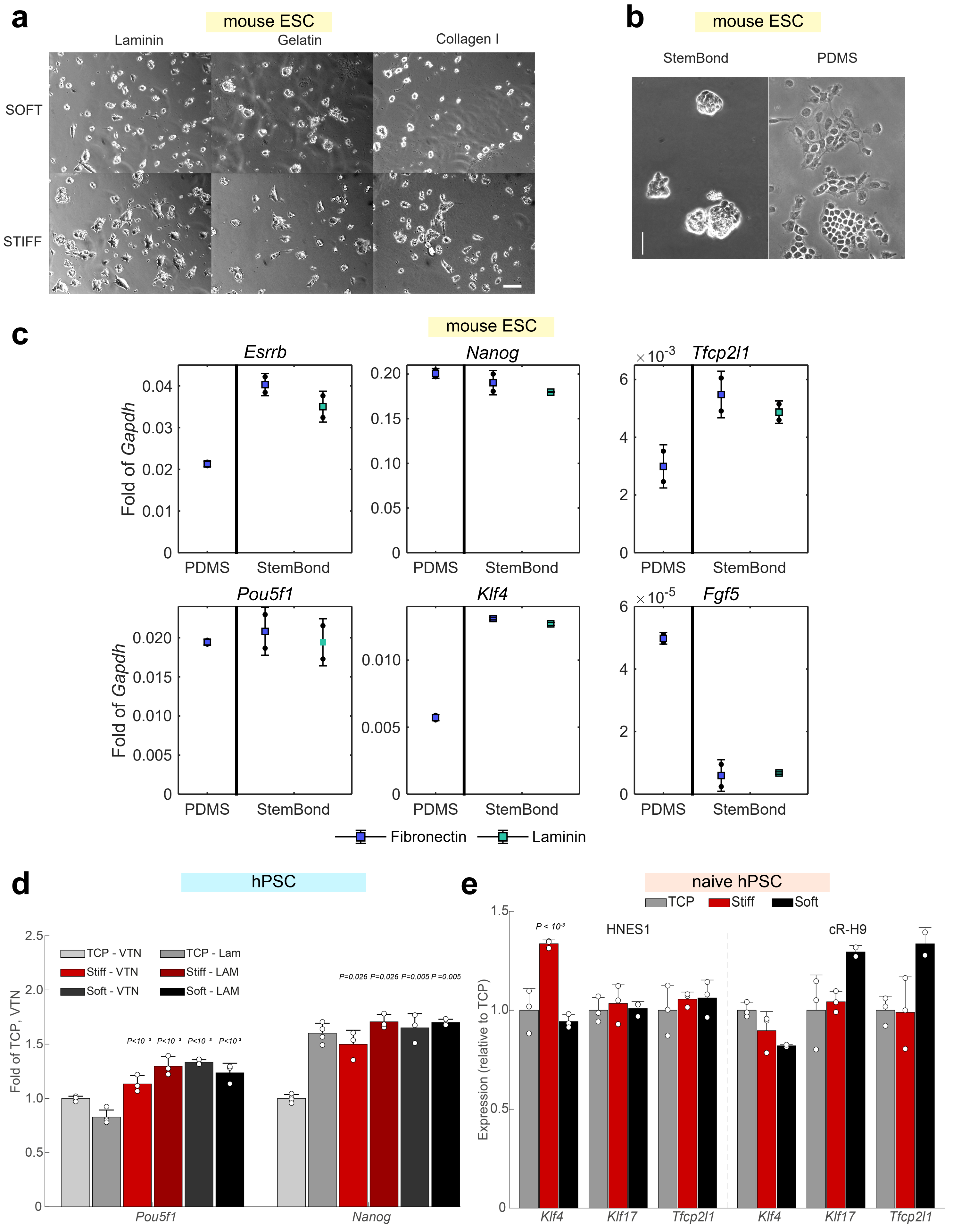

Supplementary Figure 3 (in relation to Figure 2 & 3): StemBond substrates support naïve pluripotency in mouse and human pluripotent stem cells

a – mESCs on StemBond substrates coated with different matrix proteins. Cells were in serum+LIF for 24hr. All proteins were coated with 200µg.ml^{-1} protein solution. Scale bar 100µm.

b – mESCs on commercially available soft substrates compared to soft StemBond hydrogels in serum+LIF. (Left) StemBond, mid AHA, coated with fibronectin. (Right) Ibidi Elastic Soft Substrate (PDMS) 1.5kPa coated with fibronectin following manufacturer's instruction. Scale bar: 50µm

c – Gene expression of pluripotency markers (*Esrrb, Nanog, Tfcp2l1, Pou5f1, Klf4*) and formative marker *Fgf5* on soft substrates. mESCs were cultured for 48hrs in serum+LIF. StemBond soft mid AHA hydrogels were coated with fibronectin or laminin, Ibidi soft PDMS (1.5kPa) substrates were coated with fibronectin. 'o' show values for individual samples (n = 2 independent samples) and bars show mean +/- standard deviation.

d – Mean and standard deviation of mRNA expression of *Pou5f1* and *Nanog* in hPSCs (Shef6). Cells were seeded on vitronectin and laminin-coated substrates for 4 days. n = 3, bars show standard deviation. P-values are from N-way ANOVA and indicate comparison to TCP vitronectin or TCP laminin substrates.

e - Mean and standard deviation of mRNA expression of Klf4, *Klf17* and *Tfcp2l1* in naïve hPSCs (HNES1 cell line and chemically reset naïve cells cR-H9). Cells were seeded on laminin-coated substrates for 2 days. n = 3, bars show standard deviation. P-values from one-way ANOVA are indicated when significant differences with TCP were found. Other comparisons were non-significant.

Supplementary Figure 4

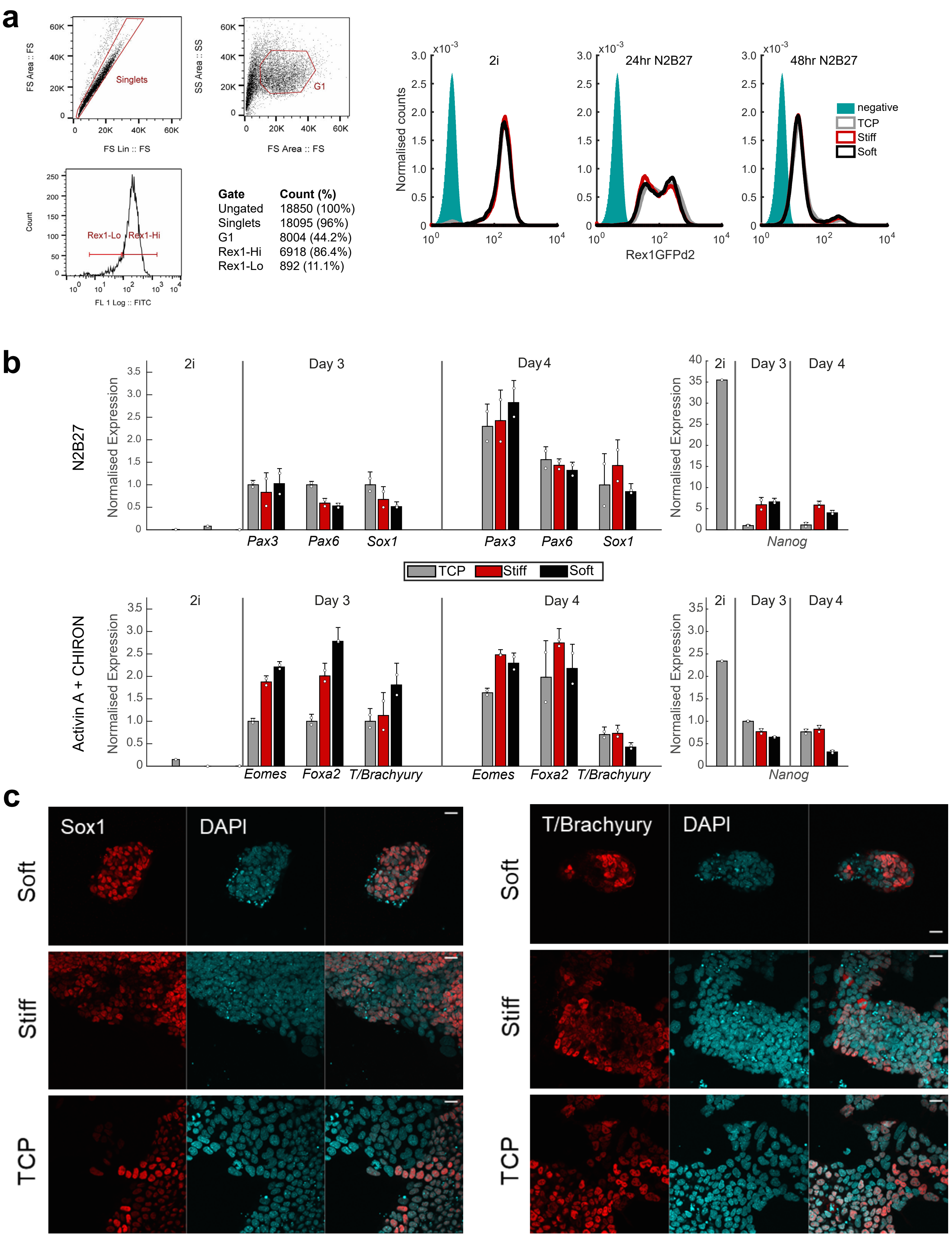

Supplementary Figure 4 (in relation to Figure 3): mESC lineage commitment is not impaired on soft and stiff substrates

a – (Left) Flow cytometry gating strategy: singlets were selected in gate “Singlets”, then debris were gated out in gate “G1”, and finally population of high and low Rex1 expressing cells were determined on the histogram. The counts and % of parent population are given for the example shown. (Right) Flow cytometry profiles of reporter line Rex1GFP::d2 cultured on TCP (grey), stiff (red) or soft (black) high AHA StemBond hydrogels for 0hr (left), 24hrs (centre) and 48hrs (right) in N2B27. The green filled histogram is a negative control. For each condition, histograms of two replicate samples were averaged and smoothed.

b – (Top) Gene expression of neuroectoderm markers *Pax3, Pax6* and *Sox1,* and naïve marker *Nanog* after differentiation in N2B27 on soft and stiff (high AHA) StemBond hydrogels, and on TCP. Expression are normalised to TCP (day 3). Note that the neural markers were very lowly expressed in 2i, and not expressed in mesoendoderm differentiation conditions (not shown). (Bottom) Gene expression of mesoendoderm markers *Eomes, Foxa2, T/Brachyury,* and naïve marker *Nanog* after differentiation in Activin A + CHIRON on soft and stiff (high AHA) StemBond hydrogels, and on TCP. Expression is normalised to TCP (day 3). Note that mesoderm markers were very lowly expressed in 2i, and not expressed in neurectoderm differentiation (not shown). Bars show mean +/- standard error (n = 2 independent samples).

c – (Left) Immunostaining for Sox1 (red) and DAPI (cyan) after 4 days. (Right) Immunostaining for T/Brachyury (red) and DAPI (cyan) in cyan after 4 days. Scale bars: 20µm.

Supplementary Figure 5

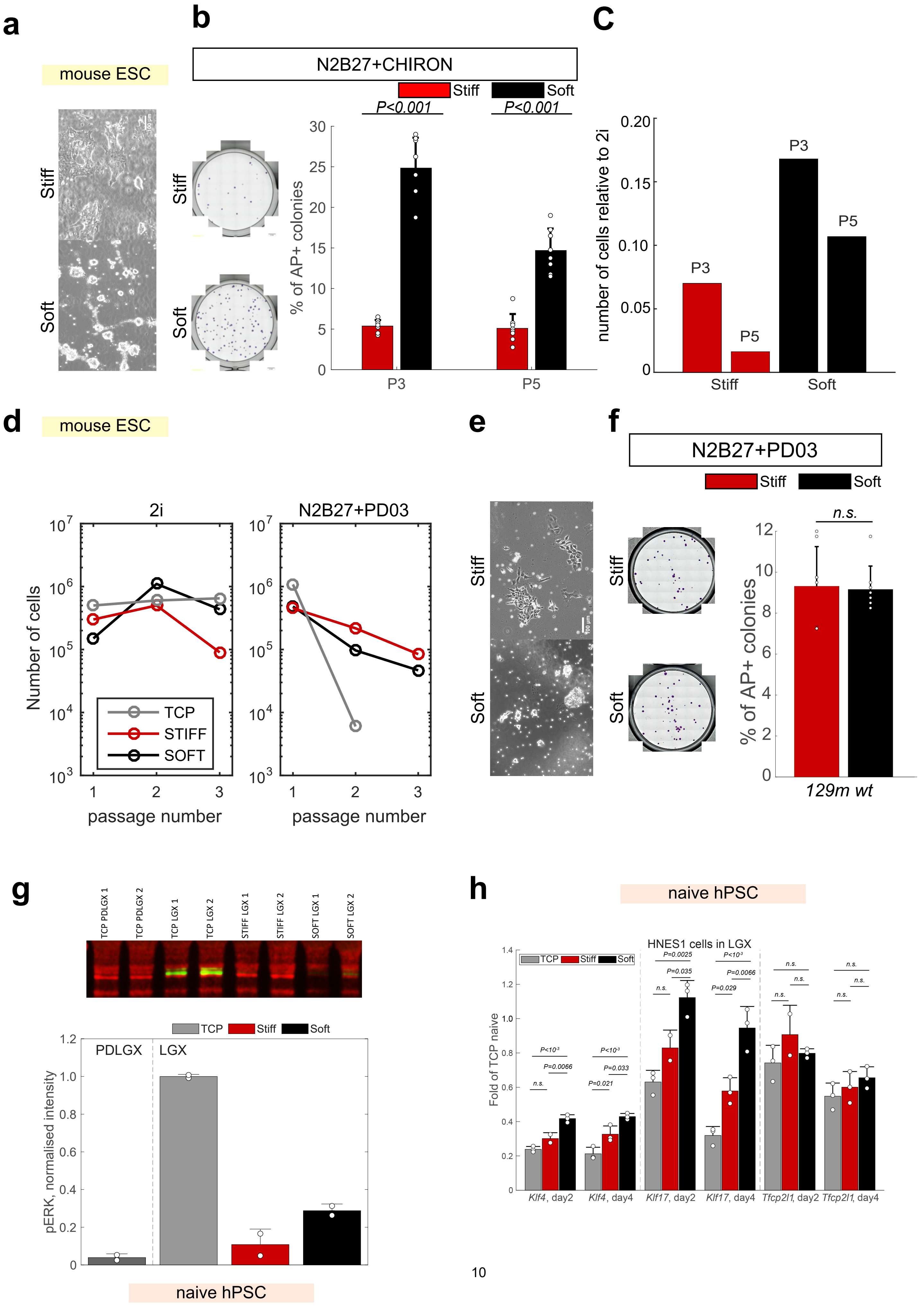

Supplementary Figure 5 (in relation to Figure 4): Soft substrates increase mouse and human naïve pluripotent stem cell self-renewal in minimal media

a – Brightfield images of mESCs on fibronectin-coated soft and stiff hydrogels after 3 passages in N2B27+CHIRON. Scale bar: 100µm

b - Clonogenicity assay after mESCs were cultured in N2B27+CHIRON following protocol (ii) (see Figure 4A). (Left) Snapshots of staining for Alkaline Phosphatase (AP) after 3 passages. (Right) Quantification of the number of AP positive colonies as % of replated cells after 3 passages (P3) and 5 passages (P5) (N=8). Error bars show standard deviation. P-values computed using a one-way ANOVA.

c – Number of mESCs counted after 3 passages (P3) and 5 passages (P5) in N2B27+CHIRON on fibronectin-coated hydrogels. The cell numbers are given relative to the number of cells counted in the control conditions (2i).

d – Number of mESCs counted at each passage in 2i, and N2B27+PD03, corresponding to Supplementary Figure 5f.

e – Brightfield images of mESCs on fibronectin-coated soft and stiff hydrogels after 3 passages in N2B27+PD03.

f - Clonogenicity assay after mESCs were cultured in N2B27+PD03 following protocol (iii) (see Figure 4a). Cell line was from 129m background. (Left) Snapshots of staining for (AP) after 3 passages. (Right) Quantification of the number of AP positive colonies after 3 passages as % of replated cells (n=8). In all panels error bars mean +/- show standard deviation and P-values indicate the outcome of an ANOVA test.

g – Quantification of pERK activity on TCP, soft and stiff StemBond hydrogels in HNES1 cells cultured in complete media PDLGX or in LGX media lacking PD03. (Top) Western Blot showing total ERK protein (red) and phospho-ERK1/2 (green). (Bottom) Mean +/- standard deviation of phospho-ERK normalised to total ERK levels. n = 2. Cells were seeded in PDLGX on laminin for 2 days before PD03 was removed. Cells were lysed 3 days after PD03 removal.

h – Expression (mean +/- standard deviation) of naïve pluripotency markers *Klf4, Klf17* and *Tfcp2l1* in HNES1 cells seeded on TCP and StemBond hydrogels. Cells were seeded in PDLGX on laminin for 2.5 days before PD03 was removed. Cells were lysed 2 and 4 days later and RNA extracted. Gene expression was measured by RT-qPCR and normalised to *Gapdh*. n = 3. P values are from one-way ANOVA. *n.s.:* non-significant.

Supplementary Figure 6

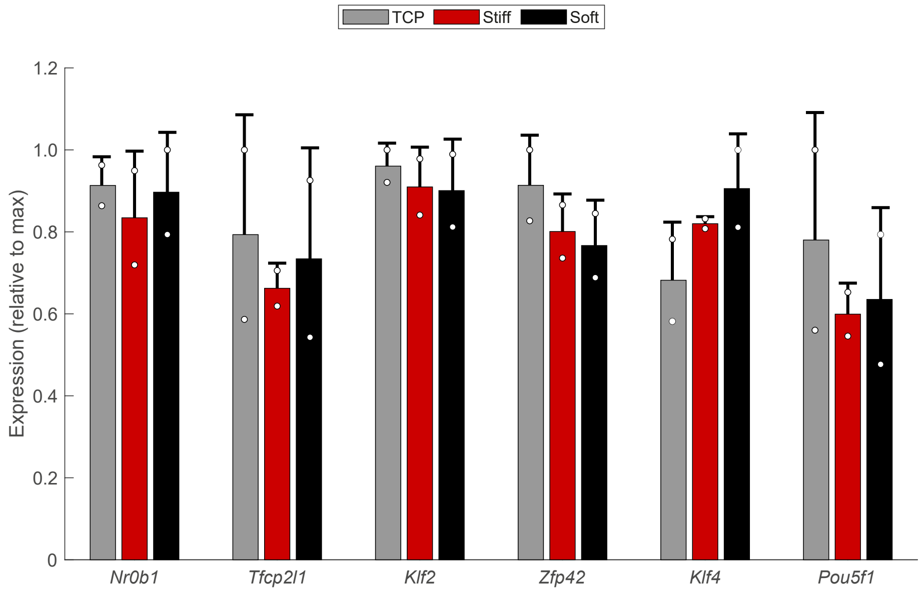

Supplementary Figure 6 (in relation to Figure 5): Reprogramming of EpiSCs is complete after 8 days on StemBond hydrogels

Expression (mean +/- std) of naïve pluripotency genes in iPSCs after 1 passage on TCP in 2i+LIF+bsd, following reprogramming on TCP, stiff or soft substrates. Expression for each replicate was normalised relative to *Gapdh* then to the highest value across all samples. n = 2 independent experiments.

Supplementary Figure 7

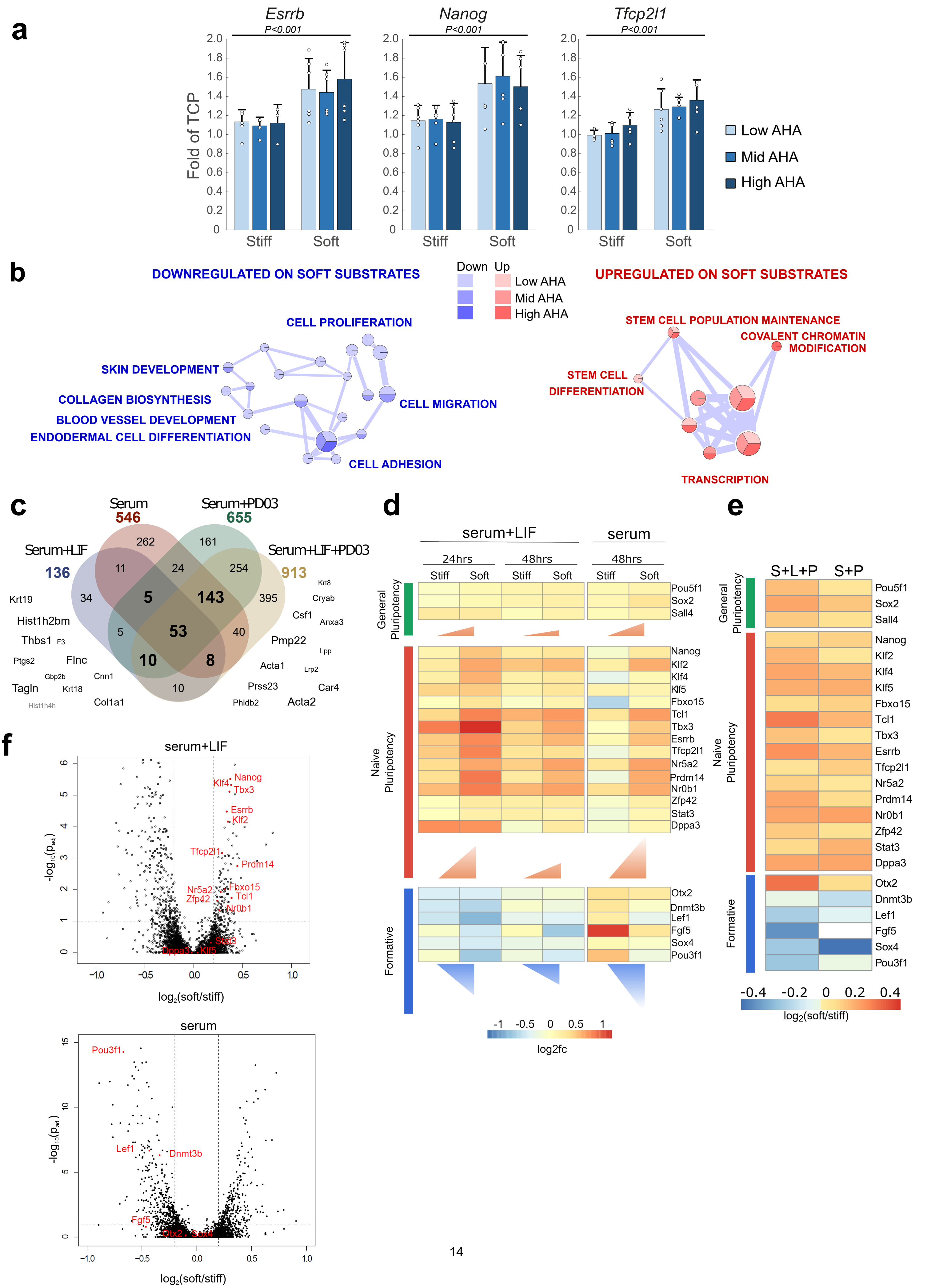

Supplementary Figure 7 (in relation to Figure 6): Differentially expressed genes and enriched pathways indicate upregulation of early embryo development pathways on soft substrates

a – Gene expression (mean +/- std) of naïve pluripotency genes *Esrrb, Nanog* and *Tfcpl21* for cells cultured for 24hr in serum+LIF on fibronectin-coated TCP soft and stiff hydrogels and low AHA (light blue), mid AHA (cyan) and high AHA (dark blue) concentrations. Expressions were normalized to TCP (N=3). P values indicate significant differences (n-way ANOVA test) due to different stiffness. AHA levels did not lead to significant differences ($P > 0.05$).

b – Network visualization of enriched biological processes due to substrate stiffness. In red are the processes enriched in upregulated genes, in blue the processes enriched in downregulated genes. Node size is proportional to the number of genes involved, and colour-charts inside each node indicate the adhesiveness for which the pathways were significantly enriched (padj < 0.1). Edge width indicates the similarity coefficient between nodes.

c - Intersection of significantly modulated genes ($p < 0.05$, abs($\log_2$fc)> 0, FPKM > 1) between soft and stiff substrates in all media conditions. In bold are the numbers of systematically modulated genes (*i.e.* in at least 3 conditions). Examples of some genes are given on the side with font size proportional to the average up/downregulation.

d – Heatmap of $\log_2$fold change of expression for a selection of general pluripotency, naïve pluripotency and formative genes. All media conditions are compared to TCP control (log2(hydrogel/TCP). The vertical side of each triangle is proportional to the average $\log_2$fc(soft/stiff) over all genes of a group for each condition.

e – Heatmap of log2(soft/stiff) for the same selection of genes as in Figure 6c and Supplementary Figure 7d. The conditions shown are serum+LIF+PD03 ("S+L+P") and serum+PD03 ("S+P").

f – (Top) Volcano plot showing significance ($-\log_{10}$(adjusted p -value)) versus fold change ($\log_2$(soft/stiff)) for the first condition shown in Supplementary Figure 7d: serum+LIF 24hrs. The dotted horizontal line shows the 10% false discovery rate level, any point above is significantly regulated. The vertical dotted lines indicate a log2 (soft/stiff) = +/- 0.2. The genes indicated in red are the naïve pluripotency markers shown in the heatmap of Figure 6c**.** (Bottom) Volcano plot showing significance ($-\log_{10}$(adjusted p -value)) versus fold change ($\log_2$(soft/stiff)) for the third condition shown in Supplementary Figure 7d: serum, 48hrs. The dotted horizontal line shows the 10% false discovery rate level, any point above is significantly regulated. The vertical dotted lines indicate a $\log_2$ (soft/stiff) = +/- 0.2. The genes indicated in red are the formative pluripotency markers shown in the heatmap of Figure 6c.

Supplementary Figure 8

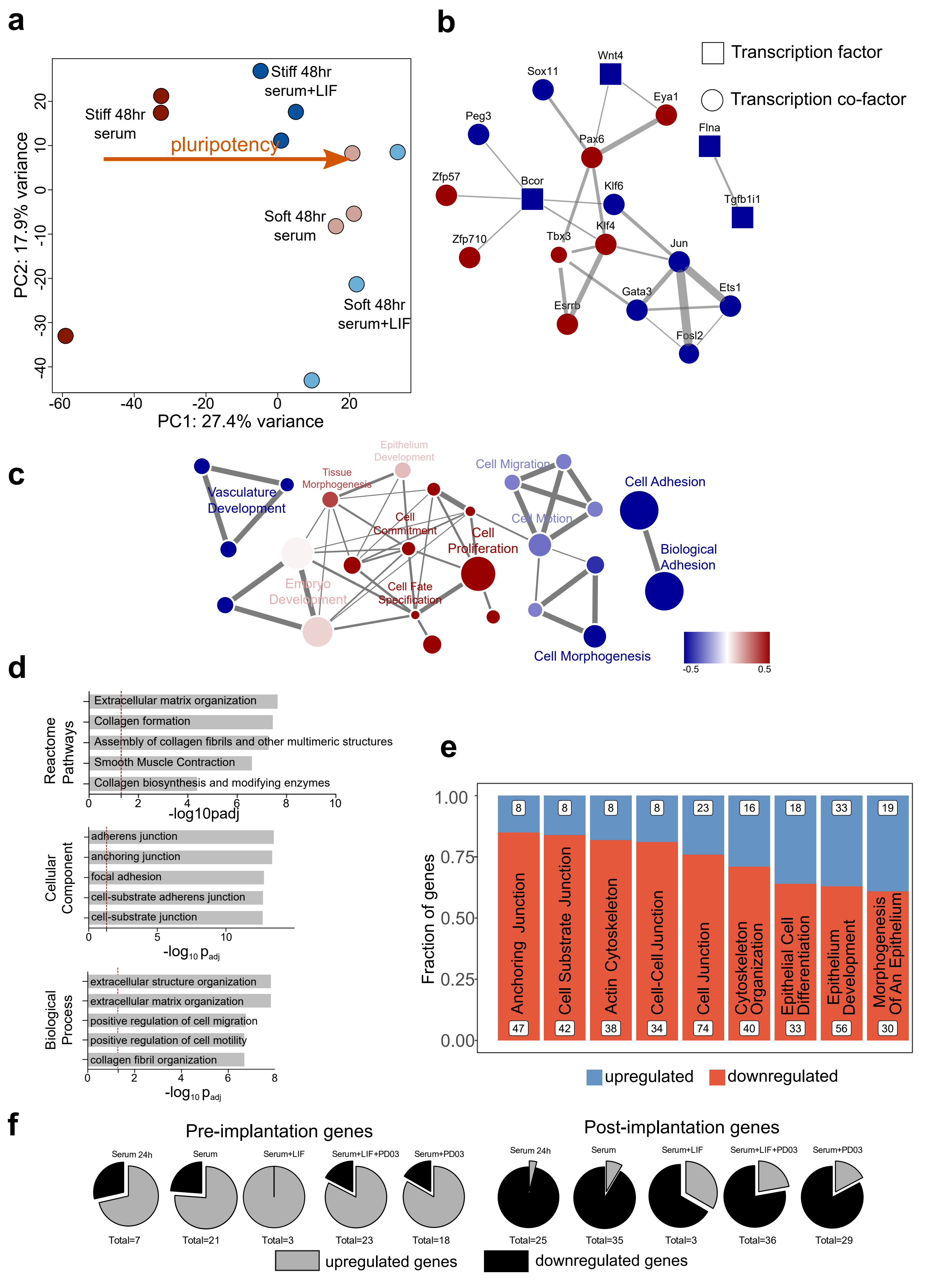

Supplementary Figure 8 (in relation to Figure 6): Naïve pluripotency and pre-implantation genes are upregulated on soft substrates across media conditions.
a - PCA plot for samples in serum+LIF and serum, 48hrs based on the highly variable genes (n=2579). The arrow indicates pluripotency potential with the more naïve pluripotent population clustered on the right-hand side.

b – Gene-Gene interaction network (https://string-db.org/) for the Transcription Factor (TF) and Transcription CoFactors (CoF) for the systematically modulated genes (n=219). Only the TF/CoF with at least 1 interaction are shown. Red / blue are for genes which are up- / down- regulated on average.

c - Top 25 enriched biological processes for genes up or downregulated in serum conditions. The size is proportional to the number of genes that are enriched in the specific process. The colour is the z-score of the percentage of up/downregulated genes for each biological process.

d – Reactome pathways as well as Cellular Components and Biological Processes that are enriched in the systematically modulated genes (bold genes in the centre of the Venn-diagram in Supplementary Figure 7c, n = 219). Dotted red lines represent the significant threshold ($p_{adj}< 0.05$).

e - Fraction of differentially expressed genes in serum which are downregulated (red) or upregulated (blue) on soft substrates for each of the selected biological processes. See also Supplementary Data File 2.

f - Proportion of significantly upregulated or downregulated genes on soft substrates compared to stiff substrates in each medium condition (in relation to Figure 6e).

Supplementary Figure 9

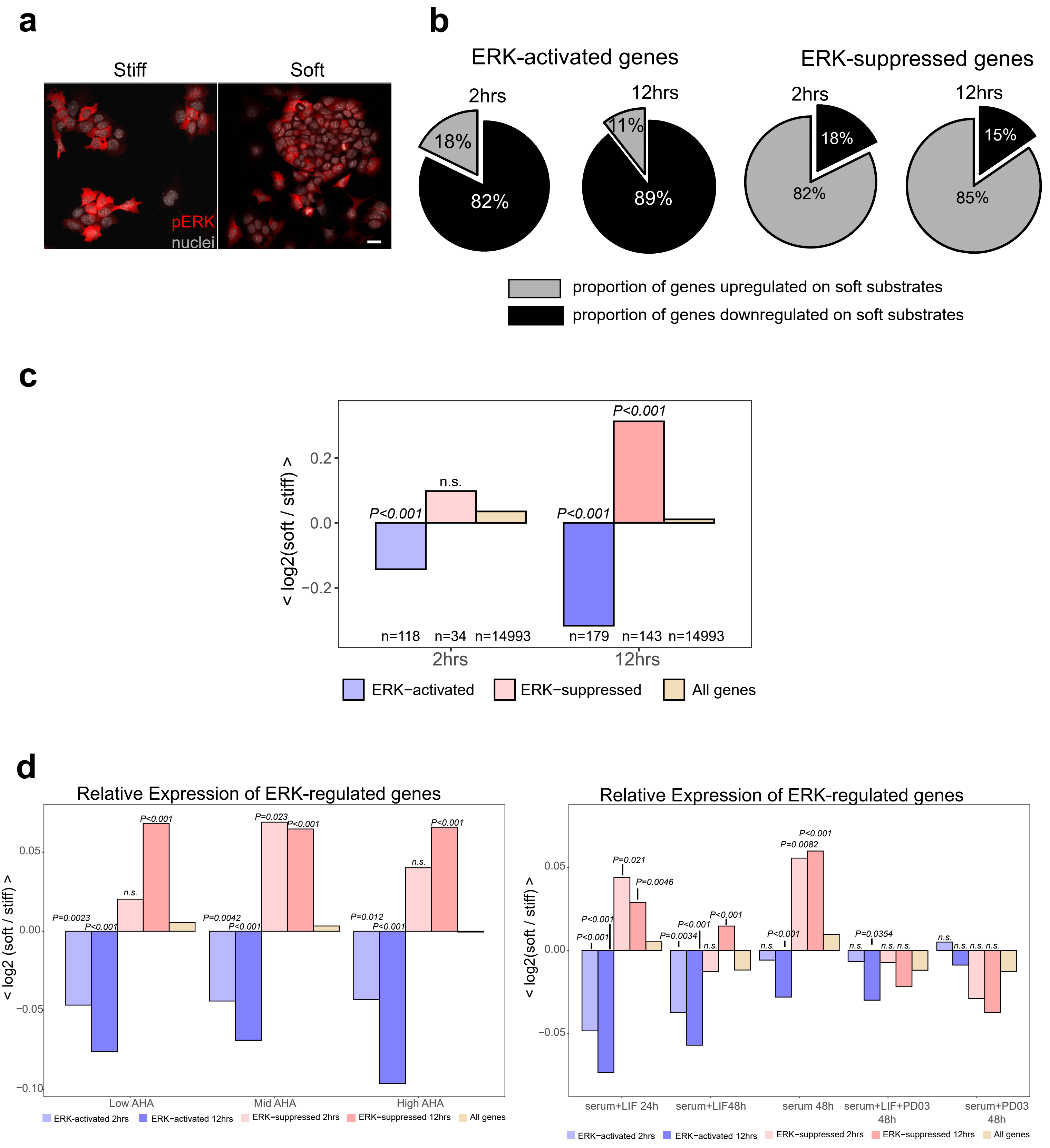

Supplementary Figure 9 (in relation to Figure 6f): ERK activity depends on substrate stiffness, but not on other environmental variables

a – Immunofluorescence for phospho-ERK (red) and DAPI (grey) on stiff (left) and soft (right) substrates. Cells were seeded on the gels for 24hrs in serum+LIF. Scale bar 20μm.

b – Pie charts giving the percentage of genes whose expression was higher on soft (grey) or on stiff (black) at 2hrs (left) and 12hrs (right) after removing PD03. Percentages are relative to all the ERK-regulated genes at 2hrs, and to the ERK-regulated genes with $|\log_2(t=12hrs/t=0hr)| > 1$ at 12hrs.

c – Average $\log_2$ fold change of expression between soft and stiff for the ERK-activated, ERK-suppressed and all expressed genes. P values obtained by a two-sided permutation test over random samples of all expressed genes. n.s.: non-significant.

d – Average fold change of expression on soft vs stiff hydrogels for ERK−targets in serum+LIF, 24hrs. ERK targets identified from PD03 removal of 2i+LIF at 2hrs or 12hrs (see Figure 6f). Fold changes for all genes (target genes and non−target genes) are shown for comparison. (Left) The expression of those targets is taken from data set of Figure 6a-b (cells seeded on different substrates). (Right) The expression of those targets is taken from data set of Figure 6c. Statistics show significant difference to the distribution of fold changes of all genes (P values computed using a one-sided permutation test), n.s.: non-significant. Statistics are computed over n = 117, 34, 176, 130 genes for each of the categories.

Supplementary Figure 10

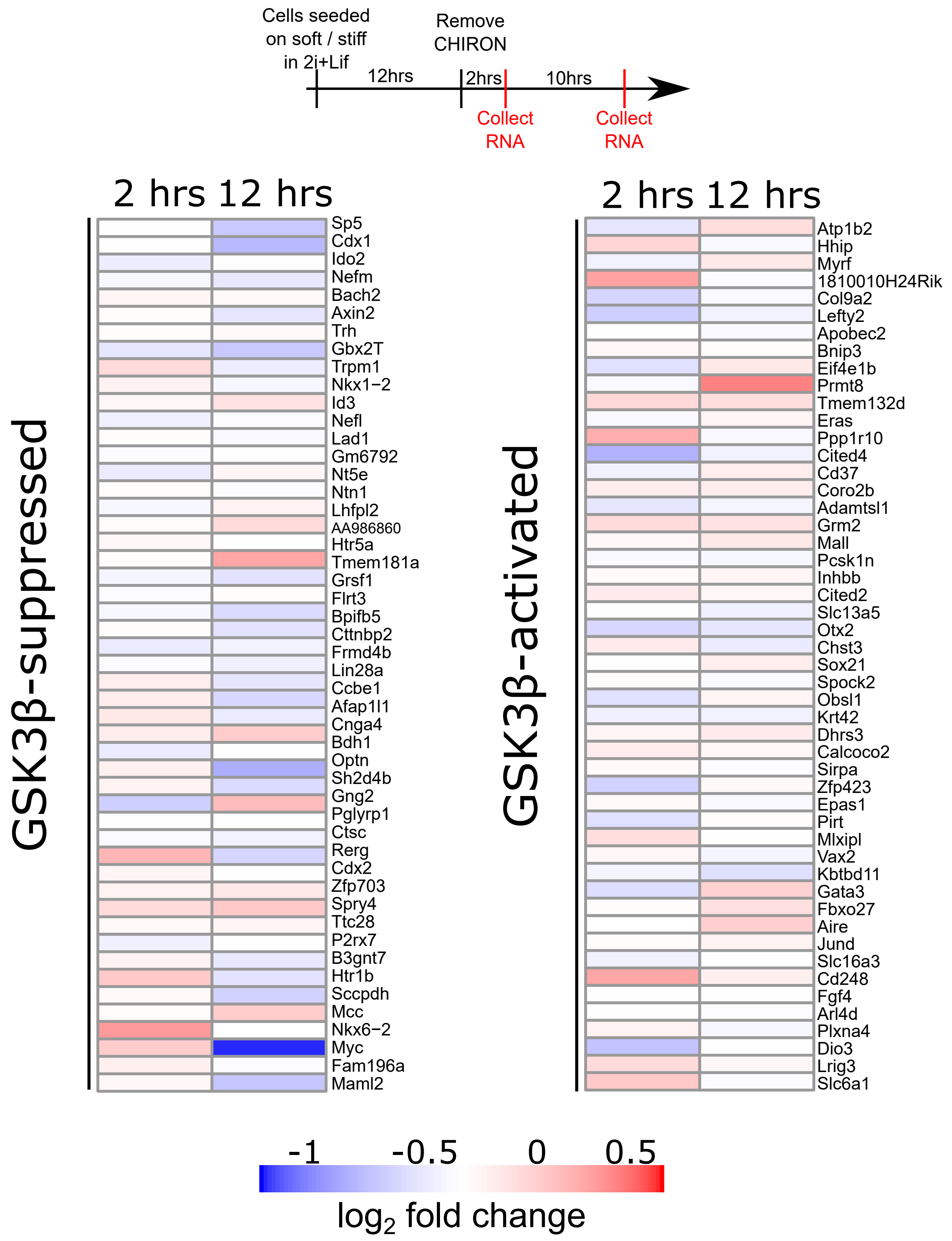

Supplementary Figure 10 (in relation to Figures 6f): GSK3β does not show any stiffness-dependent activity

(Top) Design of experiments for RNA-sequencing indicating the timing of the different steps and CHIRON removal. Experiments performed in triplicate.

(Bottom) Heatmaps of log_2 fold change in expression between soft and stiff substrates after CHIRON removal. (Left) Top 50 activated genes and (right) Top 50 suppressed genes. The genes listed are those which are co-regulated after inhibitor removal on both soft and stiff and show the largest fold change on stiff substrates when compared to control in 2i+LIF.
