# Supplementary material for: StemBond hydrogels control the mechanical microenvironment for pluripotent stem cells
Source: Nat Commun. 2021 Oct 21;12:6132. doi: 10.1038/s41467-021-26236-5 (PMC8531294; doi:10.1038/s41467-021-26236-5)
Supplement: Supplementary file 2 — Description of Additional Supplementary Files [file 41467_2021_26236_MOESM2_ESM.docx]

## Description of Additional Supplementary Files

## Title: Supplementary Data 1: Results of RNA sequencing, dataset of Figure 6A-B

Description: Normalised counts per gene for all samples that passed quality control. Outlier samples with high % of ERCC spike-in reads were discarded. Genes with less than 2 counts across all samples were discarded. Normalised counts (counts per million mapped counts) were obtained using the fpm function of DESeq2, with the robust option, i.e. using the size factors to normalise. Rows are labelled with EnsemblID, columns are labelled by sample stiffness. The samples are named by stiffness ("Soft" or "Stiff") and adhesiveness ("16" = LowAHA, "48" = MidAHA and "80" = HighAHA).

## Title: Supplementary Data 2: Results of RNA sequencing, dataset of Figure 6C-E

Description: Sheet “Table” lists the genes differentially regulated between soft and stiff substrates in different medium conditions. For each comparison, the four columns indicate mean expression (FPKM) on soft, mean expression (FPKM) on stiff, log2 fold change (soft/stiff) and adjusted p-value.
Sheet “Clusters” lists the genes belonging to the pre- and post-implantation clusters used for Figure 6E.

Sheet “Enrichment Score” lists the pathways linked to post-implantation analysed in Figure S8E (in Serum only conditions). For each pathway, the columns indicate the p-value for the pathway being significantly enriched, the enrichment score, the number of genes differentially expressed (k), the number of genes in the pathway annotation (K), the relative number of differentially expressed genes (k/K), and the ratio of number of genes downregulated on soft substrates over upregulated on soft substrates.

## Title: Supplementary Data 3: Results of RNA sequencing, dataset of Figure 6F

Description: Normalised counts for all protein coding genes for all samples during removal of PD03. Normalised counts (counts per million mapped counts) were obtained using the fpm function of DESeq2, with the robust option, i.e. using the size factors to normalise. Rows are labelled per Gene Name. Genes with no counts across all samples were discarded. Columns are labelled per sample name which indicates the substrate ("Soft"/"Stiff"), the time after PD03 removal ("t0","t12"=12hrs,"t2"=2hrs) and the replicate number (1 or 2).

## Title: Supplementary Data 4: Results of RNA sequencing, dataset of Figure S10.

Description: Normalised counts for all protein coding genes for all samples during removal of CHIRON. One sample with too low number of reads was discarded. Normalised counts (counts per million mapped counts) were obtained using the fpm function of DESeq2, with the robust option, i.e. using the size factors to normalise. Rows are labelled per Gene Name. Genes with no counts across all samples were discarded. Columns are labelled per sample name which indicates the substrate ("Soft"/"Stiff"), the time after CHIRON removal ("t0","t12"=12hrs,"t2"=2hrs) and the replicate number (1 or 2).
