# Supplementary material for: StemBond hydrogels control the mechanical microenvironment for pluripotent stem cells
Source: Nat Commun. 2021 Oct 21;12:6132. doi: 10.1038/s41467-021-26236-5 (PMC8531294; doi:10.1038/s41467-021-26236-5)
Supplement: Supplementary file 7 — Reporting Summary [file 41467_2021_26236_MOESM7_ESM.pdf]

nature portfolio

Corresponding author(s): Kevin J Chalut

Last updated by author(s): Sep 7, 2021

Reporting Summary

Nature Portfolio wishes to improve the reproducibility of the work that we publish. This form provides structure for consistency and transparency in reporting. For further information on Nature Portfolio policies, see our Editorial Policies and the Editorial Policy Checklist.

Statistics

For all statistical analyses, confirm that the following items are present in the figure legend, table legend, main text, or Methods section.

n/a	Confirmed	
☐	☒	The exact sample size (*n*) for each experimental group/condition, given as a discrete number and unit of measurement
☐	☒	A statement on whether measurements were taken from distinct samples or whether the same sample was measured repeatedly
☐	☒	The statistical test(s) used AND whether they are one- or two-sided<br>*Only common tests should be described solely by name; describe more complex techniques in the Methods section.*
☐	☒	A description of all covariates tested
☐	☒	A description of any assumptions or corrections, such as tests of normality and adjustment for multiple comparisons
☐	☒	A full description of the statistical parameters including central tendency (e.g. means) or other basic estimates (e.g. regression coefficient) AND variation (e.g. standard deviation) or associated estimates of uncertainty (e.g. confidence intervals)
☐	☒	For null hypothesis testing, the test statistic (e.g. *F*, *t*, *r*) with confidence intervals, effect sizes, degrees of freedom and *P* value noted<br>*Give P values as exact values whenever suitable.*
☒	☐	For Bayesian analysis, information on the choice of priors and Markov chain Monte Carlo settings
☒	☐	For hierarchical and complex designs, identification of the appropriate level for tests and full reporting of outcomes
☒	☐	Estimates of effect sizes (e.g. Cohen's *d*, Pearson's *r*), indicating how they were calculated

Our web collection on statistics for biologists contains articles on many of the points above.

Software and code

Policy information about availability of computer code

Data collection	RNA was sequenced using Illumina HiSeq 4000. Flow cytometry data was collected using Summit v4.3.02 (Beckman-Coulter). Confocal imaging performed using Zeiss ZEN Blue or Leica LAS X software (version unknown). AFM performed using JPK SPM Processing Software v6.1.86 & v6.1.131. RT-qPCR performed on StepOne Plus (Life Technologies, unknown version)
Data analysis	AFM: JPK SPM Processing Software v6.1.86 & v6.1.131;<br>RNA-seq data: Trim Galore v 0.4.4_dev; GSNAP v 2015-09-29; HTSeq 0.6.1; STAR 2.5.2a; Tophat v 2.1.0; Featurecount vl.5.0; R packages: DESeq, DESeq2, FactoMineR; Cytoscape v3.7.0 with Enrichment Map Plugin; DAVID 6.7 an 6.8; STRING;<br>Flow Cytometry: FlowJO v9.3.3;<br>RT-qPCR: qPCR data processed with StepOne v2.3.<br>Images: ImageJ 1.52i.<br>All graphs: Matlab 2015b;

For manuscripts utilizing custom algorithms or software that are central to the research but not yet described in published literature, software must be made available to editors and reviewers. We strongly encourage code deposition in a community repository (e.g. GitHub). See the Nature Portfolio guidelines for submitting code & software for further information.

Data

Policy information about availability of data

All manuscripts must include a data availability statement. This statement should provide the following information, where applicable:

- Accession codes, unique identifiers, or web links for publicly available datasets
- A description of any restrictions on data availability
- For clinical datasets or third party data, please ensure that the statement adheres to our policy

All RNA-sequencing data generated in this study (corresponding to Figures 6A-B, 6C-E and 6F as well as S7-S10) have been deposited in the GEO database under accession code GSE125617 (https://www.ncbi.nlm.nih.gov/geo/query/acc.cgi?acc=GSE125617). The data generated in this study underlying Figures 1C-D-E-F, 2A-C, 3A-B-C-D, 4B-C-D, 5C-D and Supplementary Figures S1B-C-D-E, S2B-C-E-F, S3C-D-E, S4B, S5B-C-D-F-G-H, S6, S7A, S8E-F, and S9B-C-D are provided as a Source Data file.

Field-specific reporting

Please select the one below that is the best fit for your research. If you are not sure, read the appropriate sections before making your selection.

[x] Life sciences [ ] Behavioural & social sciences [ ] Ecological, evolutionary & environmental sciences

For a reference copy of the document with all sections, see nature.com/documents/nr-reporting-summary-flat.pdf

Life sciences study design

All studies must disclose on these points even when the disclosure is negative.

Sample size	No statistical method was used to predetermine sample size. We repeated experiments on multiple independent biological repeats (independent experiments indicated in figure legends) and/or on several gel replicates (independent samples indicated in figure legends).
Data exclusions	In figure 6A-B (RNAsequencing) sample 7 was removed for analysis because of the high percentage of ERCC spike-in counts (> 1%)
Replication	Gene expression assays (RT-qPCR) were done with 2 or 3 technical replicates per sample.
Randomization	For image analysis of clonogenicity assays, image names were blinded and processed in a random order to avoid experimenter bias. Colonies were then manually counted after automatic thresholding. In other analyses (image analysis, RT-qPCR, WB, RNAseq), automatic quantification was used.
Blinding	To analyse the clonogenicity assays, sample info was removed from image names before manual counting the number of colonies per image. In other analyses, quantification was done by automated software, removing experimenter bias, therefore not requiring blinding

Reporting for specific materials, systems and methods

We require information from authors about some types of materials, experimental systems and methods used in many studies. Here, indicate whether each material, system or method listed is relevant to your study. If you are not sure if a list item applies to your research, read the appropriate section before selecting a response.

Materials & experimental systems

n/a	Involved in the study	
[ ]	[x]	Antibodies
[ ]	[x]	Eukaryotic cell lines
[x]	[ ]	Palaeontology and archaeology
[ ]	[x]	Animals and other organisms
[x]	[ ]	Human research participants
[x]	[ ]	Clinical data
[x]	[ ]	Dual use research of concern

Methods

n/a	Involved in the study	
[x]	[ ]	ChIP-seq
[ ]	[x]	Flow cytometry
[x]	[ ]	MRI-based neuroimaging

Antibodies

Antibodies used	Primary Antibodies<br>Phospho-p44/42 MAPK (Erk1/2) (Thr202/Tyr204), 4370, Cell Signaling #17 Ref:05/2016, #17 Ref:01/2017, 1:1000 (WB), 1:200 (IF)<br>p44/42 MAPK (Erk1/2) (137F5) Rabbit mAb, 4695 S, Cell Signaling, #21 Ref:05/2016, 1:1000 (WB)<br>Phospho-Stat3 (Tyr705) (M9C6) Mouse mAb, 4113, Cell Signaling, #5 Ref:07/2016, 1:1000 (WB)<br>Phospho-Stat3 (Tyr705) (D3A7) XP® Rabbit mAB, 9145, Cell Signaling, #22 Ref:12/2013, 1:1000 (WB)

Stat3 (124H6) Mouse mAb, 9139 S, Cell Signaling, #10, Ref:12/2016, 1:1000 (WB)
Gapdh (D4C6R) Mouse mAb, 97166 S Cell Signaling #3 Ref:05/2017, 1:2000 (WB)
Gapdh (D16H11) XP® Rabbit mAb, 5174 S Cell Signaling #6 Ref:11/2016, 1:2000 (WB)
Esrrb Monoclonal Mouse IgG2a Clone # H6705 (m), PP-H6705 Perseus Proteomics, Lot A2, 1:500 (WB)
Nanog (rabbit, poyclonal), A300-398A, Bethyl Laboratories, Inc., 1:2000 (WB)
H3 (rabbit, poyclonal), ab1791, Abcam, 1:5000 (WB)
Integrin beta-1 / CD 29 Clone 9EG7 (RUO) (rat), 553715, BD Biosciences, 1:100 (IF)
Sox1 (rabbit, polyclonal), 4194S, Cell Signaling, #2 Ref:03/2019, 1:100 (IF)
T/Brachyury (goat, polyclonal), #AF2085, R&D Systems, Lot KQP0315041, 1:400 (IF)
Fibronectin [EPR19241-46] (rabbit), ab199056, Abcam, 1:1000 (IF)
Fibronectin (rabbit, polyclonal), ab2413, Abcam, 200µg/ml (AFM), 1:500 (IF)
Normal Rabbit IgG (rabbit, polyclonal), 2729 S, Cell Signaling, 200µg/ml (AFM)
Phospho-Paxillin (Tyr118) Antibody, 2541 Cell Signaling, #6 Ref:08/2015, 1 :50 (IF)
Phospho-p44/42 MAPK (Erk1/2) (Thr202/Tyr204) (E10) Mouse mAb, 9106, Cell Signaling, 1:1000 (IF)

Secondary Antibodies
Anti-rabbit IgG, HRP-linked, 7074 S, Cell Signaling, #26 Ref:12/2016, 1:2500 (WB)
Anti-mouse IgG, HRP-linked, 7076 S, Cell Signaling, #32 Ref:12/2015, 1:2500 (WB)
Alexa Fluor®555 Phalloidin, 8953, Cell Signaling, #3 Ref:11/2016, 1 :20 (IF)
Goat anti rabbit 680, A21109, Thermofisher, 1:20000 (WB)
Goat anti mouse 790, A11357, ThermoFisher, 1:20000 (WB)

Validation

Antibodies were validated by the supplier for species reactivity (mouse, human) and specificity of the target. We reproduce some of the information here and refer the reader to the datasheets for further details.

Phospho-p44/42, Cat 4370: detects endogenous levels of p44 and p42 MAP Kinase (Erk1 and Erk2) when dually phosphorylated at Thr202 and Tyr204 of Erk1 (Thr185 and Tyr187 of Erk2), and singly phosphorylated at Thr202. This antibody does not cross-react with the corresponding phosphorylated residues of either JNK/SAPK or p38 MAP kinases. Reacts with Human, Mouse and other species (supplier information). We also conducted a negative control with MEK inhibitor which showed absence of phosphorylated ERK but presence of total ERK.

ERK, Cat 4695 S: detects endogenous levels of total p44/42 MAP kinase (Erk1/Erk2) protein. The antibody does not cross-react with JNK/SAPK or p38 MAP kinase. Reacts with Human, Mouse and other species (supplier information).

phospho Stat3, Cat 4113: Phospho-Stat3 (Tyr705) (M9C6) Mouse mAb detects endogenous levels of Stat3 only when phosphorylated at Tyr705. This antibody does not cross-react with phospho-EGFR or the corresponding phospho-tyrosines of other Stat proteins. Reacts with Mouse, Human and other species (supplier information). We conducted a negative control in a sample without LIF which showed absence of phosphorylated Stat3 but presence of total Stat3.

phospho Stat3, Cat 9145: Phospho-Stat3 (Tyr705) (D3A7) XP® Rabbit mAb detects endogenous levels of Stat3 only when phosphorylated at tyrosine 705. This antibody does not cross-react with phospho-EGFR or the corresponding phospho-tyrosines of other Stat proteins. Reacts with Mouse, Human and other species (supplier information). We conducted a negative control in a sample without LIF which showed absence of phosphorylated Stat3 but presence of total Stat3.

Stat3, Cat 9139: Stat3 (124H6) Mouse mAb detects endogenous levels of total Stat3 protein. Reacts with Huma, Mouse and other species (supplier information).

Gapdh, Cat 97166: GAPDH (D4C6R) Mouse mAb recognizes endogenous levels of total GAPDH protein. Reacts with mouse, human and other species.

Gapdh, Cat 5174: GAPDH (D16H11) XP® Rabbit mAb detects endogenous levels of total GAPDH protein. Reacts with mouse, human and other species (supplier information).

Esrrb, Cat PP-H6705: Reacts with Human, mouse (supplier information). We controlled that this antibody gave no signal in a cell line (mouse embryonic fibroblast) that did not express Esrrb, as seen in Figure 2b.

Nanog, Cat A300-398A: Reacts with mouse (supplier information). We controlled that this antibody gave no signal in a cell line (mouse embryonic fibroblast) that did not express Nanog, as seen in Figure 2b.
H3, ab1791: Based only on sequence homology, we expect the antibody to react with multiple variants of H3 such as H3.1, H3.2 and H3.3. Reacts with mouse, human and other species (supplier information).

Integrin beta-1 / CD 29, Cat 553715: The 9EG7 antibody reacts with the 130-kDa integrin β1 chain (CD29). CD29 has a broad tissue distribution, including lymphocytes, endothelia, smooth muscle, and epithelia. 9EG7 mAb has been shown to inhibit both the α6β1-mediated binding of lymphocytes to endothelial cells and the adhesion mediated by activated, but not unactivated, α4β1-integrin. The source of the immunogen was mouse lymph node-derived endothelial cell line TME. Reacts with mouse (supplier information).

Sox1, Cat 4194: Sox1 Antibody detects endogenous levels of total Sox1 protein. It does not cross-react with endogenous levels of other Sox proteins, including Sox2 and Sox3. Reacts with mouse and rat, predicted to react with human (supplier information).

T/Brachyury, Cat AF2085: Detects human Brachyury in direct ELISAs and Western blots. In direct ELISAs, less than 10% cross-reactivity with recombinant human TBX 6 is observed. Reacts with mouse, human.

Fibronectin, Cat ab199056: Reacts with mouse, rat. Does not react with human. Western blot and IHC tests of human samples were negative (supplier information). This antibody was used so as not to detect human plasma fibronectin or bovine rhodamine fibronectin used for coating substrates.

Fibronectin, Cat ab2413: Reacts with mouse, human. Positive Control WB: Human colon tissue lysate, HepG2, NIH 3T3 whole cell lysate. IHC-P: Human kidney tissue. ICC: HeLa cells (supplier information). This antibody was used to stain for human plasma fibronectin used to coat the hydrogels.

IgG, Cat 2729: Normal Rabbit IgG is an unconjugated rabbit polyclonal antibody that is routinely used as a non-specific IgG control (supplier information).

Phospho-Paxillin (Tyr118), Cat 2541: Phospho-Paxillin (Tyr118) Antibody detects endogenous levels of paxillin only when phosphorylated at tyrosine 118. The antibody does not cross-react with other tyrosine phosphorylated proteins. Reacts with mouse, human and other species (supplier information)

Phospho-p44/42 MAPK (Erk1/2), Cat 9106: Phospho-p44/42 MAPK (Erk1/2) (Thr202/Tyr204) (E10) Mouse mAb detects endogenous levels of p44 and p42 MAP Kinase (Erk1 and Erk2) when dually phosphorylated at Thr202 and Tyr204 of Erk1 (Thr185 and Tyr187 of Erk2), but not singly phosphorylated at Thr202 or Tyr204. This antibody does not cross-react with the corresponding phosphorylated residues of either SAPK/JNK or p38 MAP kinase. This antibody may cross-react with an unknown cytoskeletal protein in some cell lines as visualized by immunofluorescence. Reacts with human, mouse and other species. (supplier information).

Eukaryotic cell lines

Policy information about cell lines

Cell line source(s)

E14Tg2a mouse embryonic cells are a subclone of the E14 mouse cell line (Hooper et al., Nature 1987). The subclones were derived in the groups of Austin Smith and Jenny Nichols.
Rex1GFPd2 were derived on a E14 background by the group of Austin Smith (Kalkan et al., Development 2017).
Rosa26-CreERT2+/+ mouse embryonic stem cells from a C57BL/6-Agouti background were derived by the group of BK Koo.
HNES1 human naive embryonic stem cell line were w derived by the groups of Austin Smith and Jenny Nichols (Guo et al., Stem Cell Reports, 2016,https://doi.org/10.1016/j.stemcr.2016.02.005)
cR-H9 (chemically reset) human naive embryonic stem cell lined were derived by the group of Austin Smith from H9 cells obtained from the WiCell Research Institute (Guo et al., Development, 2017, https://doi.org/10.1242/dev.146811)
Shef-6 human pluripotent stem cell line was obtained from the Austin Smith group and was originally derived at Sheffield University (Canham et al., 2015)
All cell lines derived in the Smith, Nichols or Koo groups at University of Cambridge were generously donated to us for this work.

Authentication

No authentication procedure performed by us for this study. Authentication was performed previously by the groups deriving the cell lines, see references above.

Mycoplasma contamination

All cell cultures tested negative for mycoplasma contamination. Testing was performed regularly.

Commonly misidentified lines (See ICLAC register)

No commonly misidentified cell lines were used in this study.

Animals and other organisms

Policy information about studies involving animals; ARRIVE guidelines recommended for reporting animal research

Laboratory animals

Mus musculus were used for chimaera experiments:
Species: mouse
Strain: C57BL/6 for host embryos and stud males
Strain: CBAB6F1 for recipient females
Strain: CD1 for the vasectomised males
Males were between 3 and 12 months old, females are over 6 weeks, and up to 6 months.

Wild animals

None used in this study

Field-collected samples

None used in this study

Ethics oversight

This research has been regulated under the Animals (Scientific Procedures) Act 1986 Amendment Regulations 2012 following ethical review by the University of Cambridge Animal Welfare and Ethical Review Body (AWERB). Use of animals in this project was approved by the ethical review committee for the University of Cambridge, and relevant Home Office licenses (Project license No. 80/2597) are in place.

Note that full information on the approval of the study protocol must also be provided in the manuscript.

Flow Cytometry

Plots

Confirm that:

- [x] The axis labels state the marker and fluorochrome used (e.g. CD4-FITC).
- [x] The axis scales are clearly visible. Include numbers along axes only for bottom left plot of group (a 'group' is an analysis of identical markers).
- [x] All plots are contour plots with outliers or pseudocolor plots.
- [x] A numerical value for number of cells or percentage (with statistics) is provided.

Methodology

Sample preparation	Cells were detached, pelleted and resuspended in PBS and placed on ice. They were processed in the flow cytometer within 30 minutes.
Instrument	Dako Cytomation CyAn ADP high-performance unit
Software	Collection: Summit v 4.3.02 (Beckman-Coulter) Analysis: Flowjo v9.3.3 (gating) and Matlab 2015b (plotting and statistics)
Cell population abundance	No cell sorting in this study.
Gating strategy	Data was first gated on Forward Scatter (Line vs Area) to eliminate doublets, then further gated on the forward and side scatter to eliminate debris. Finally, population of Rex1-Hi and Rex1-Lo were determined based on the histogram of Rex1GFPd2 expression. Data was continuously acquired to obtain at least 4000 cells within the G1 gate.

- [x] Tick this box to confirm that a figure exemplifying the gating strategy is provided in the Supplementary Information.
